# IDMBD: Intelligent Diagnostic Modelling of Bipolar Disorder at its early onset

**DOI:** 10.1038/s41598-026-57662-4

**Published:** 2026-06-13

**Authors:** K. A. Yashaswini, S. Kokila, Arunkumar Balakrishnan, K. Madhura, S. Anbukkarasi, Raghavendra M. Devadas

**Affiliations:** Manipal Institute of Technology Bengaluru, Manipal Academy of Higher Education, Manipal, India

**Keywords:** Bipolar disorder, Machine learning, Feature, Deep neural network, Mobile computing device, Forecasting, Engineering, Mathematics and computing

## Abstract

The proposed study presents an Intelligent Diagnostic Modelling of Bipolar Disorder (IDMBD), which represents an underlying framework operating on a Mobile Computing Device (MCD). The approach is meant to acquire multi-modal heterogeneous features in order to profile the participants more effectively with the highest granularity. IDMBD adopts a standard scale for forecasting the criticality state of Bipolar Disorder (BD). The feature-based profiling is carried out based on five discrete attributes collected by MCD, which, after undergoing a series of analytical processing, is subjected to a deep neural network-based learning model. An extensive evaluation is being carried out in multiple combinations of both machine learning and deep learning models to find that IDMBD exhibits optimal performance when integrated with Random Forest and Decision Tree with respect to multiple performance metrics.

## Introduction

Bipolar Disorder (BD) is characterised by various medical conditions related to mental health, which sometimes are quite challenging to differentiate and distinguish from other mental disorders^1–3^. The majority of existing screening approaches of BD are based on questionnaire-based tools as well as an interview process that results in either image, video, or text-related information^4–6^, and sometimes it also includes radiological data too^7^. All the screening methods are based much time consuming that demands multiple visits to the doctor. However, early detection of BD is necessary for enhanced treatment outcomes, prevention of disability, minimised threat of comorbidities, better prognosis, etc^8^. Machine Learning (ML) has recently contributed towards addressing multiple ranges of problems associated with mental health disorders^9–15^. However, there is still an unsolved challenge too. Data associated with BD could be very scarce, especially in early phases of BD, while it can also be inconsistent, too, which is definitely not enough to build any training model that eventually lacks diversity^16^. There is often a cyclical pattern of mood for an individual with BD, which could be quite complex, too, posing enough challenge for a standardised model to carry out forecasting^17^. Finding the progression point of BD is another bigger challenge. From a practical viewpoint, the prominent impediment also resides in integration ML based approach with clinical settings^18^. Adoption of Artificial Intelligence (AI) in sensitive medical information is something that many healthcare professionals may be reluctant to use for diagnosis, especially when there is no ground-breaking, proven approach to date.

Hence, the proposed study presents a novel and cost-effective solution to address the ongoing challenges associated with early detection of BD. This paper presents a novel multimodal feature promoting and enriching the profiling of the normal and BD participants for better identification of indicators using unique intelligent modelling.

The proposed computational model depicts a risk screening tool that is essentially meant for leveraging early monitoring rather than any form of definitive system for clinical diagnosis. Although behavioral screening performed by an AI, it is essential to realize that proposed model depicts an explanatory model assessed on cohort that is relatively of limited size performed on highly controlled research environment. Hence, the accomplished outcome should be understood as universal generalizability or an evidence of clinical validation. The key motive of IDMBD is towards analyzing and exploring the possibility towards early screening of BD adopting multimodal behavioral analytics. Yet, the study considers validating with large-scale clinical environment as next phase of future work direction. The novelty of this paper is that it presents a cost-effective framework that doesn’t demand exclusive resources or platforms or any extra cost towards deployment or maintenance, harnessing a hybrid form of an innovative learning strategy different from existing solutions in the present times.

The fundamental novelty of the proposed Intelligent Diagnostic Modelling of Bipolar Disorder (IDMBD) lies in its unified integration of multimodal behavioural sensing, adaptive feature-level fusion, and resource-aware hybrid learning within a mobile-centric framework. Unlike existing approaches that predominantly rely on single-modality data or computationally intensive Deep Learning (DL) pipelines, IDMBD introduces a lightweight yet scalable architecture that simultaneously leverages heterogeneous inputs such as positional data, sleep cycles, emotional states, self-assessment scores, and hybrid media signals.

From a technical standpoint, IDMBD differs from existing multimodal BD detection systems in three key aspects. First, it employs a *framework archive strategy* that dynamically selects predictive models based on available feature modalities, thereby effectively handling missing data without relying on imputation. Second, it introduces a structured aggregation of statistically derived and deep-learned features, enabling better representation of temporal and behavioural variability. Third, the system is explicitly designed for deployment on mobile computing devices, ensuring real-time feasibility and cost-effectiveness, which is often overlooked in prior studies that depend heavily on high-resource environments.

Furthermore, the proposed hybridisation of Random Forest (RF) and Decision Tree (DT) is theoretically motivated by their complementary strengths. RF offers robustness through ensemble learning and variance reduction via bagging, while DT provides interpretability and efficient rule-based decision boundaries. When integrated within the IDMBD framework, RF contributes to improved generalisation across heterogeneous feature spaces, whereas DT facilitates local decision optimisation and transparency. This combination is particularly suitable for multimodal behavioural data, where feature interactions are complex, non-linear, and often partially correlated. Hence, the hybrid RF + DT configuration achieves a balance between predictive accuracy, computational efficiency, and interpretability, making it well-suited for early-stage BD detection in resource-constrained environments. Different from classical frameworks of predicting BD that highly depend upon either fusion of static features, or on DL models with resource consumption, or standalone modality, the proposed model presents an integrated architecture that collaborates adaptive selection of framework with multimodal sensing of behavior. The prime distinction of proposed architecture is related to dynamic selection of predictive model based on available modality and not depending upon classical imputation of missing-value. Apart from this, different from previous methodologies that specifically offers more importance to predictive accuracy, proposed model optimizes resource feasibility, interpretability, and predictive performance jointly towards implementing standard devices of mobile computing without any demands towards using specialized hardware or wearable sensors. The collaboration of behavioral multimodal analytics with lightweight hybrid learning depicts key unique contribution of the proposed model.

It is essential to consider that proposed model is anticipated to work as clinical decision-support system or an early risk assessment model and it should not be considered as a definitive platform of diagnosis for BD. The resultant yielded by the model is especially meant to help the clinician by providing them information related to behavioral risk that is obtained from observations taken from multimodal mobile centric sources. Therefore, the proposed framework is interpreted as a supportive tool of computation that compliments the procedures of clinical assessment and it is not meant currently for substituting formal medical diagnosis or psychiatric evaluation.

The organisation of the manuscript is as follows: In  “Related work” section presents a discussion of existing studies, while the discussion of research methodology is carried out in “Research method” section. The design and implementation are described in “Design implementation” section. In “Results and discussion” section illustrates the result accomplished, while the conclusion is stated in “Conclusion” section.

## Related work

This section is a continuation of our prior research work^19^. It has been noted that Random Forest (RF) has been used in the form of both a standalone approach^20^ as well as ensemble approaches too, towards the detection of BD^21^. RF is also reportedly used for classifying BD from a normal individual using specific media input^22^. Support Vector Machine (SVM) is the next frequently adopted learning approach towards predicting mental health^23^, early prediction of depression integrated with Multi-layered Perceptron^24^, and multimodal imaging-based BD detection^25^. Another ML approach, Naïve Bayes (NB), has been witnessed in literature not much as a standalone method but mainly used in an ensemble method for classifying BD from other personality disorders^26^, study of clinical data and neuroimaging towards prediction^27^, and motor activity-based depression detection^28^. Logistic Regression (LoR) has been increasingly used in the study of mental disorders, where some of the recent studies show some notable contributions towards solving the prediction problem using gene expression^29^, used as a benchmark method for comparisons^30^, and based predictive modelling^31^. Linear Regression (LiR) has been reportedly used for investigating mental disorders using neuroimaging^32^ while it was also used for predicting mood^33^. The K-Nearest Neighbour (KNN) model is another frequently used model, mainly in the form of an ensemble algorithm and not much as a standalone algorithm in existing studies towards the detection of BD^34^. Finally, current studies have also witnessed the usage of Decision Tree (DT) towards differentiating BD from other potential disorders^35–37^, characterisation of emotion^38^, solving detection problem^39^. At the same time, there are evolving studies of DL where Convolution Neural Network (CNN) and Long Short-Term Memory (LSTM) have been used for similar purposes^40–42^. Hence, this is the biggest challenge between machine learning-based approaches and newly evolved DL-based approaches, where from resource-viewpoint ML always support cost effective deployment. However, from a complexity viewpoint, DL serves towards optimal performance. Table 1 highlights the comparative positioning of proposed model with existing studies.

**Table 1:** Difference between IDMBD and Existing Studies.

Study characteristics	Proposed IDMBD	Existing studies
Focus on early-stage BD screening	Primary objective	Partial
Real-time feasibility on standard smartphones	Supported	Limited
Behavioural + emotional + positional fusion	Yes	Rarely integrated together
Lightweight resource-aware design	Explicitly considered	Limited attention
Hybrid ML-DL integration	Unified integration	Partially explored
Handling missing modalities	Framework archive-based adaptive selection	Mainly imputation-based
Dependency on wearable/specialized hardware	No	Frequently required
Mobile-centric deployment	Yes	Rarely emphasized
Multimodal behavioural integration	Yes	Limited and partially explored
Single-modality analysis	No	Commonly adopted

The above information of comparison between proposed system and existing approaches showcases that proposed model offers some essential novel features towards bridging the gap among adaptive predictive modelling, lightweight mobile deployment, and behavioral analytics towards screening BD in early stages. The next section presents a new model which hybridizes both deep and ML towards early detection of BD.

## Research method

**Fig. 1:**
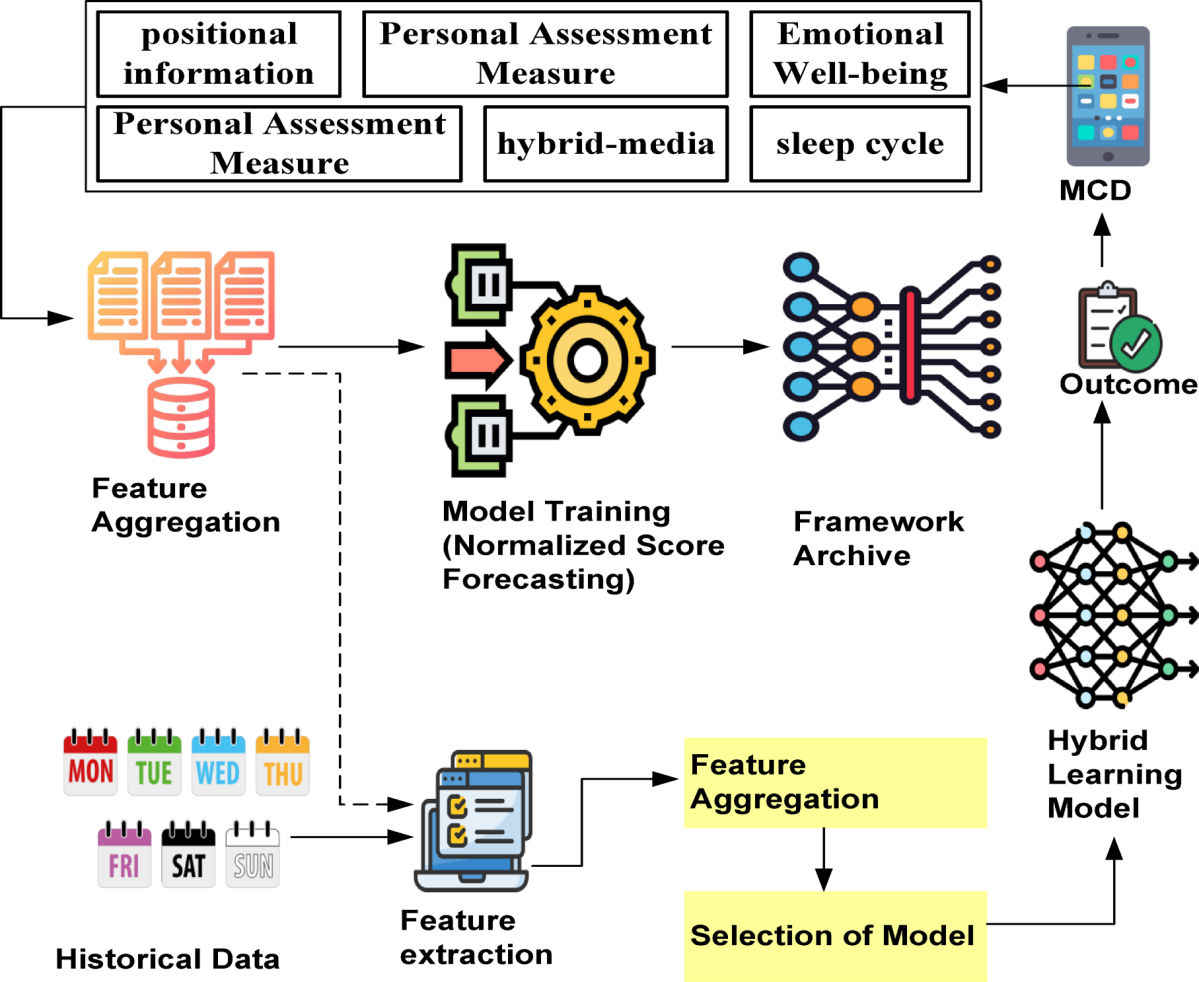
Architecture of IDMBD.

The prime purpose of the proposed study is to design and develop a novel and lightweight early-predictive framework that is capable of determining an early stage of BD, along with differentiating BD from a normal individual. The complete framework is targeted to function in the form of a Mobile Computing Device (MCD) on normal cellular services. Any iOS or Android-based cellular phone can actually host this framework. There are no other dependencies for using any form of wearables or any other software or hardware to actually use the proposed study framework. The device is responsible for collecting the necessary information, which, when forwarded to its underlying design framework that is actually hosted in cloud services, will perform prediction. Further, it should be noted that MCD will be solely capable of performing a complete operation, while its dependency on the cloud is only required to update the trained dataset for better predictive efficiency. The architecture of the proposed system, known as Intelligent Diagnostic Modelling of Bipolar Disorder (IDMBD), is shown in Fig. 1. The architecture in Fig. 1 also showcases the interaction between local processing within cloud-assisted analytical operation and mobile computing device. Hence it exhibits the lightweight strategy of deployment implemented by proposed model towards monitoring BD in practical environment.

According to Fig. 1, it can be noted that IDMBD works on a specific design flow, which is discussed as follows:

- **Usage of Framework**: The proposed model of IDMBD acts as an application framework in MCD, which doesn’t require its users to wear any wearables or need any specific skills to use it. The framework demands that its user to enrol themselves by stating their personal information. The framework also passively collects location information of the user to be used for predictive diagnosis of BD. Further, the active collection of information is carried out by prompting the user to manually feed (i) hybrid media contents using speech signal, text content, and video, (ii) actual state of emotion in 24 h, (iii) duration of sleep, and (iv) emotional state assessed by participants. All these four modes of active input to the system is terms as digital profiling. However, it is again essential to understand that complete input from the participants is based on multiple optional clicks on the user interface. All this information is then processed locally to generate an outcome, while the outcome is further cross-checked by connecting it online cloud server.
- **Data Collection**: All the collected data are instantly fed to the core framework of IDMBD within the MCD in order to perform local processing. However, for global processing, the data needs to be forwarded to a cloud server using either cellular data or using hotspots. Apart from the listed types of necessary information, no other form of background data is collected in this process.
- **Intelligent Profiling**: The proposed system of IDMBD extracts multiple numbers and types of features that are further appended together. The integrated features are then subjected to a series of model training with multiple features, which leads to the generation of a complete framework archive. During the testing phase, the extracted features are used towards historical data, followed by applying a hybrid ML algorithm in order to generate the final forecasting.

Apart from this, it is essential to understand that the proposed IDMBD framework targets developing a simplified and yet innovative concept of using multi-modal information. Such multi-modal information doesn’t require specialised skill, nor does it demand any expensive processing design stage or any specialized dedicate transmission services. A simple MCD with normal cellular service with a mobile data pack is sufficient to operate this framework with full-fledged functionalities. One important consideration of the IDMBD framework is that it hypothesises that there are various root causes as well as trigger points of BD, and hence, there is a need to initiate a design framework where multiple modalities of information can be used to yield enriched features, unlike any existing system. Powered by ML, this framework will also open up more scope for investigation with cost-effective deployment strategies towards early detection of BD. For the sake of reproducibility and clarity, the complete implementation work of IDMBD is summarised as follows:

- The system initiates the acquisition of multi-modal data originating from the mobile devices.
- The raw data is then subjected to preprocessing, starting from filtering missing values, followed by normalisation and aggregation of the normalised data.
- The system extracts features from multiple modalities, viz., location, scales of assessment, emotion, media, and sleep.
- Both statistically computed feature as well as positional data is subjected to spatial clustering.
- The feature combinations are constructed, followed by archiving the framework.
- The system initiates a rating estimation model using DNN.
- Prediction is then carried out using a hybridisation of RF, DT, and SVM.
- Multiple performance metric has been used.

It is to be noted that the extraction of media features can be carried out by a complex form of DL models, which are usually executed in a resourceful cloud backend. On the contrary, lightweight data acquisition as well as preprocessing operation is carried out by the mobile device. Both primary sensing and acquisition of data can be simplified using smartphones or mobile computing devices. The proposed scheme uses a tailored mobile application for acquiring active forms of data (e.g. sleep logs, video, audio, text, etc.) as well as passive forms of data (usage behaviour, location, etc.). Various forms of preprocessing operations were carried out locally on raw data, followed by transmitting them securely to the analytic server. The proposed hybridisation scheme facilitates feasibility towards smartphones that have constrained resources. The next section illustrates the complete design implementation aspect of IDMBD.

## Design implementation

The complete design of the proposed study model is carried out by collecting variability of mobility/location, patterns and duration of sleep, expression of facial video, speech recordings, and logs of textual mood. All these indicators are potentially influenced by behavioural changes as well as mood instability during bipolar disorder. The primary ideology of the IDMBD is towards presenting a robust schema that is capable of assessing the status of BD for given computed profiled information of a patient. The considered learning architecture of IDMBD consists of a training and testing stage, where the former stage is considered to play a critical role. The complete training operation involves the acquisition of potential features, feature aggregation, a rating estimation framework, and building a predictive model. Different profiled data are acquired for obtaining different variants of features, followed by building various combinations to arrive at a forecasting model of normalised scores. Hence, diversified information related to BD can be captured by IDMBD. Different combination of features was used for training each model, while the framework archive is constructed by combining all the trained models. Finally, the IDMBD selects the best model on the basis of the forecasted normalised score from the complete framework archive. Further, IDMBD extracts features from historical profiled information that are further clustered together on the basis of multiple combinations of features. In the final implementation stage, the forecasted outcome originating from all models is integrated, while the state of BD is evaluated using the outcome of the forecasted normalised score.

In order to offer clarity on implementation and readability, the workflow of proposed model can be broadly classified into four discrete stages. The first stage begins with acquisition of multimodal data obtained from devices of mobile computing while the second stage begins with performing preprocessing operation and extraction of features related to behavioral aspects. The third stage initiates DL models towards aggregation and estimation of ratings while fourth stage performs integration of decision and prediction using hybrid ML model. The illustration of each design component of IDMBD is as follows:

### Acquisition of potential features

**Fig. 2:**
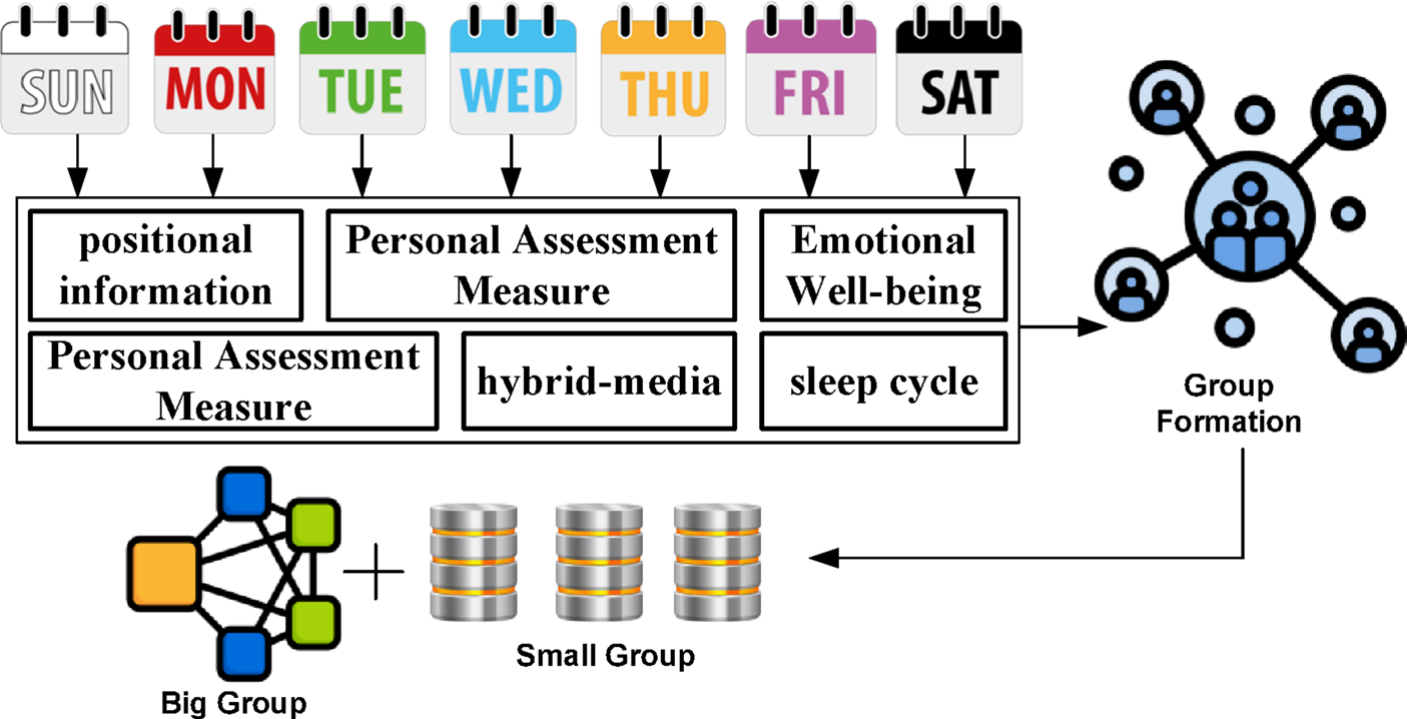
Feature Acquisition.

A multi-modal feature extraction scheme has been presented in IDMBD in order to offer more granular acquisition of information for early prediction of BD. For this purpose, IDMBD carry out acquisition of potential features from the following: (i) positional information, (ii) Personal Assessment Measure, (iii) Emotional Well-being, (iv) hybrid-media, (v) sleep cycle. The primary step in this process is to consider the quantity of missing data for each class of information, followed by either dropping or retaining the information. This operation not only resists imprecise training operations and consequences, but it can also control the influence of an increased ratio of missing information. Figure 2 pictorially showcases the acquisition of potential features. The workflow diagram exhibited in Fig. 2 shows the process of sequential transformation of inputs related to heterogeneous behaviour into structural multimodal features that are aggregated towards supporting predictive learning subsequently.

The following are further elaborated illustrations of the feature extraction process:

- **Positional Information-based Feature Acquisition**: Inspired by the existing study model^40^, IDMBD considers a specific number of features associated with positional information based on longitude and latitude data linked to each smaller group. The features considered for this purpose are group cardinality (A_1_), information dispersion (A_2_), adjusted information dispersion (A_3_), geographical variability (A_4_), residence duration (A_5_), movement duration (A_6_), total travel span (A_7_), daily motion (A_8_), daily motion on standardized coordinates (A_9_), and daily motion relative to home (A_10_). Further, the proposed scheme assesses various levels of activities exhibited by the participants, considering the quantity of clusters for depicting locations or activities that engage them in 24 h. IDMBD uses clustering operation based on spatial concentration of group densities^43^, which increases data point density upon identifying the stationary position of participants at particular coordinates. Hence, data point density minimises with the movement of participants. The operation of the initial mode of feature acquisition is based on multiple activity-related attribute inclusions. The first feature group cardinality (A_1_) is obtained by applying^43^, depicting a distinct number of cluster locations with a specific number of minimum data points (say 20 data points) for generating groups. The second feature, information dispersion (A_2_), is empirically computed as:

1 $$\:{A}_{2}=\sum\:_{i=1}^{N}{\alpha\:}_{i}\:$$

In the above expression (1), the computation of information dispersion (A_2_) is carried out considering α_i_ representing the product of outcome probability prob and the logarithm of the same probability log(prob) with base 2. The core motive of expression (1) is to understand the variability score of participants in different *N* groups. Increased value of A_2_ will represents even distribution of the duration of participant on multiple groups and vice versa. The third feature adjusted information dispersion (A_3_) is obtained from A_2_ itself by further dividing them with quantity of groups, i.e. A_3_ = A_2_/log_e_(N). It is to be noted that adjusted information dispersion (A_3_) is more linked to understanding the distribution of the group. Upon finding A_3_ = 1, it represents an even distribution of data points, while A_3_ = 0 represents complete data points residing within the same group. The fourth feature, geographical variability (A_4_), defines the fluctuation position of participation. It is mathematically computed as:

2 $${\mathrm{A}}_{{\mathrm{4}}} = {\text{ log }}\left( {{\mathrm{SD}}_{{\mathrm{1}}} + {\mathrm{SD}}_{{\mathrm{2}}} } \right)$$

In the above expression (2), the computation of geographical variability (A_4_) is carried out by computing the standard deviation SD_1_ associated with longitude and the standard deviation SD_2_ associated with latitude. A closer look into the mathematical expression of (2) showcases us-age of the logarithmic function, which is mainly due to addressing the artefacts that could be possibly present in data. Eventually, the log is used for compensating for any statistically evolving unwanted kurtosis or skewness associated with the region of distribution of data points. The fifth feature is residence duration (A_5_), which represents the proportion of duration that the participant is found to be within their residence. The idea is to understand that a higher score of A_5_ will represents lesser physical activities. The calculation of this feature is carried out by obtaining all the data points between sleep time and wake-up time for efficient classification of groups. Further, the duration associated with the cumulative data points group at residence is divided by 24 h in order to finally obtain A_5_. The sixth feature, movement duration (A_6_), represents the overall time when the participant is found to be non-stationary. This feature is calculated by adding the duration attribute for all the data points that do not reside in any groups while clustering was performed, and this final duration score is divided by 25 h to obtain the A_6_ score.

The seventh feature, total travel span (A_7_), represents the overall spatial distance travelled by the participant that is acquired by adding up all the distances residing between any two data points where these data points further depicts level of physical activity. The mathematical representation of A_7_ is as follows:

3 $$A_{7} = \mathop \sum \limits_{{i = n}}^{N} \sqrt {\Delta x^{2} + \Delta y^{2} }$$

In the above-expression (3), the computation of total travel span (A_7_) is carried out by a simplified distance equation where Δx represents the difference of current data point xi and next data point x_i−1_ on latitudinal-plane while Δy represents the difference of current data point y_i_ and next data point y_i−1_ on longitudinal-plane. The lower and higher values of suffix *i* are *n* and *N*, representing a small to a larger number of data points. The eight-feature daily motion (A_8_) represents the positional order of a participant over a complete 24 h. IDMBD uses a statistical methodology towards identifying the periodic signal featured by inconsistent temporal data using least-squared method from positional information^44^. Further, the study uses daily motion on standardised coordinates (A_9_) for addressing possibilities of false positives. The mathematical computation of A_8_ is as follows:

4 $${\mathrm{A}}_{{\mathrm{8}}} = {\mathrm{log}}\left( {\Sigma \mu {\mathrm{n}}} \right)$$

In the above-expression (4), the computation of daily motion (A_8_) is carried out by applying a logarithmic function to the summed up values of *µ*,* which* represents the energy score obtained by dividing the frequency at a specific data point by the difference of two frequencies at two different hours of the day. Considering *i* = 2 in Eq. (4), µ_n_ = 2 will represent µ_1_ and µ_2_ as energy score at latitude and longitude, respectively. The term energy can be computed using the power spectral density. Hence, computation of standardised coordinates (A_9_) is carried out considering the same Eq. (4), only with the difference that 0 and 1 are considered as mean and variance score for normalising the data points linked to longitude and latitude, respectively. Finally, the last feature, daily motion relative to home (A_10_), is used for overcoming the possibility of information loss from A_8_ and A_9_ due to the adoption of the power spectral density score at latitude and longitude. Hence, daily motion relative to home (A_10_) performs the computation of the distance of resident grouping and standardises with 0 and 1 as the mean and variance for distance normalisation. Hence, a better accuracy score is achieved towards monitoring all natural and internal processes for participants.

**Fig. 3:**
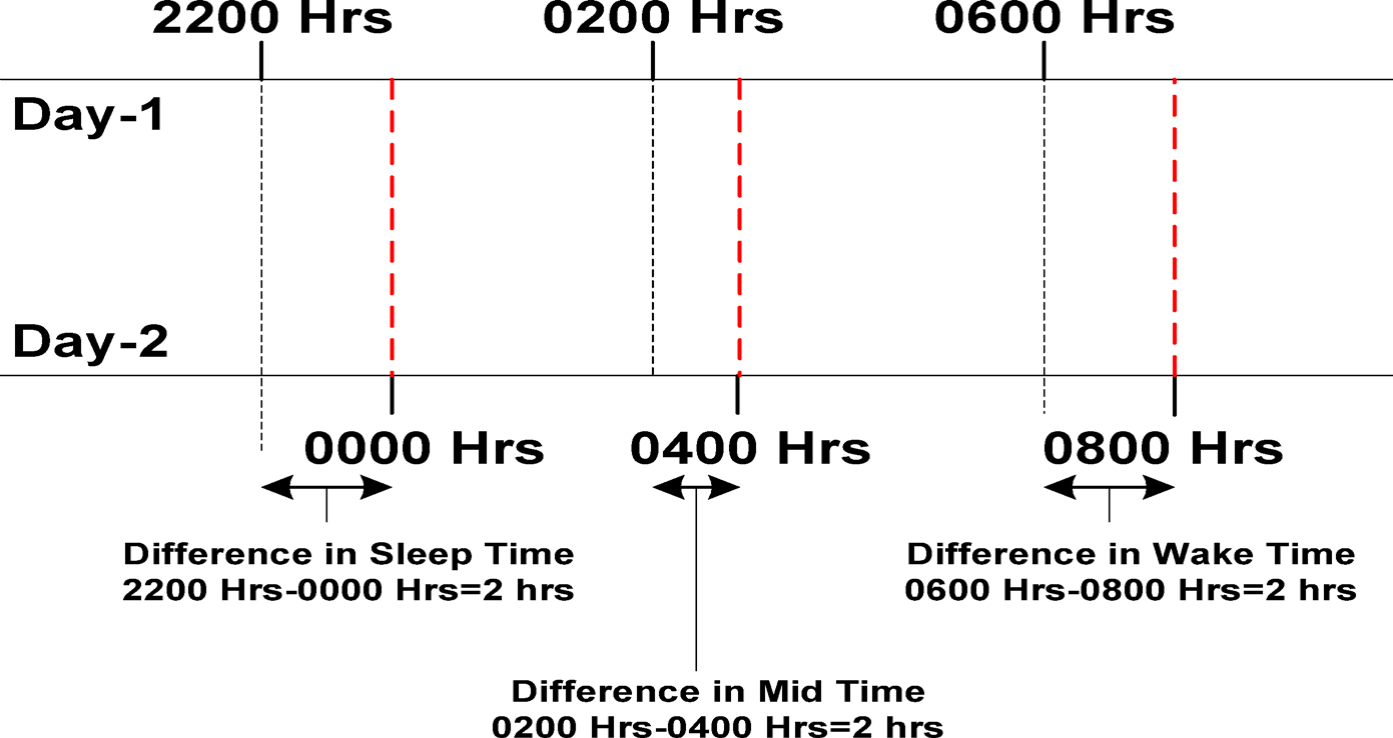
Understanding Sleep Cycle Attributes.

- **Personal Assessment Measure-based Feature Acquisition**: This is the second type of feature adopted in the proposed study, where a standardised assessment tool of the Depression, Anxiety, and Stress Scale – 21^45^ and the Altman Self-Rating Mania Scale^46^ have been used. Both of these consist of questions carrying a rating, which is meant for self-assessment of the mental state associated with the participant. The feature is extracted from the scores obtained as an outcome of the standardised tool.
- **Emotional Well-being-based Feature Acquisition**: The proposed scheme extracts information about the type of features associated with the emotional well-being of a participant regularly. For a given subgroup of data, the system extracts values of features based on each 24-hour state of the emotional state of participants. This is followed by a mean calculation for arriving at the trend of participants’ mood, along with their respective standard deviation. Therefore, each 24-hour data point associated with the state of mood of the participant is statistically captured in this part of the feature.
- **Hybrid media-based Feature Acquisition**: The term hybrid media will refer to digitised content obtained from various multimedia in-formation either in personal settings or in clinical settings. IDMBD uses primary and secondary mood attributes obtained from this hybrid media content. The primary mood attribute is characterised by emotional states that are self-declared by the participants themselves, followed by statistical computation of mean and standard deviation towards each mood attribute. The secondary mood attribute is obtained by applying standard models of sentiment recognition. The values associated with each mood type are deployed in the form of scores for secondary mood attributes. Finally, all the scores of hybrid data are added up, followed by statistical computation of the mean and standard deviation. The hybrid media data consists of video data, speech signals, and text data, where the proposed IDMBD uses the BERT model^47^ for text modality, semi-CNN for speech modality^48^, and CNN-RNN for video modality^49^. The accuracy is eventually found to be more for textual content (~ 88%) and much less for video content (~ 57%), while speech data has medium-range accuracy (~ 66%).
- **Sleep Cycle-based Feature Acquisition**: The study also considers three different types of sleep cycle correlating with the possible early prediction of BD, such as sleep time span, sleep halfway point, and sleep cycle consistency. The first parameter, sleep time span, is defined as the duration between the person fall sleep to wake up with full consciousness. The second parameter sleep halfway point, is defined as the mean duration between the state of sleeping and complete wake-up. The third parameter, sleep cycle consistency, is defined as consistency in the sleeping cycle observed for any person for consecutive two observation days. The statistical mean and standard deviation are computed for all the above three types of sleep cycle attributes. Pictorially, it is shown in Fig. 3 for better understanding.

An illustrative discussion of temporal variation in sleep as well as their degree of influence on obtaining consistency features of sleep-cycle connected with behavioral irregularities is shown in Fig. 3. According to Fig. 3, consider waking up in the morning at 0600 h and sleeping in 2200 h, which will mean a sleep time span of 8 h. Further, in second day, consider the same person who now sleeps at 1200 h and wakes up at 0800 h. In this condition, the sleep halfway point for the first day is at 0200 h, while for the second day sleep halfway point is at 0400 h. So, sleep time gap for first and second day is st_gap_=2 h, and wake-up time gap for first and second day is wt_gap_=2 h. Hence, sleep cycle consistency (s_con_) is calculated as:

5 $$\:{s}_{con}=\frac{24-\left|\right({st}_{gap}-{wt}_{gap}\left)\right|}{24}$$

From the above mathematical expression (5), the sleep cycle consistency is now easier to find. The idea is to consider sleep and its different types of cycles as one of the indicators to affect the mental health of the participants. There is a potential correlation between multimodal behaviour signals and mood disorders. Robustness can be enhanced by integrating heterogeneous signals while minimising dependencies towards single modality.

### Feature aggregation

**Fig. 4:**
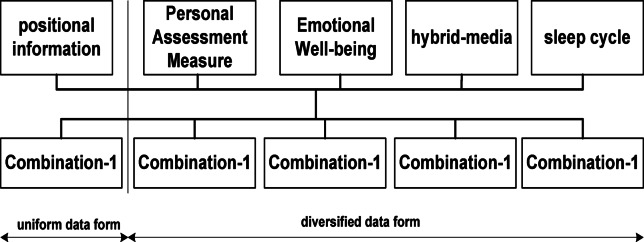
Feature Aggregation.

All the features that have been accumulated are subjected to aggregation before being subjected to predictive operation. It should be noted that there is no explicit conventional feature selection technique applied apart from the feature extraction method discussed in prior section. All five types of features (i) positional information, ii) Personal Assessment Measure, iii) Emotional Well-being, iv) hybrid-media, and v) sleep cycle have been considered for this purpose. Figure 4 showcases 5 possible combinations of uniform data form and diversified data form.

According to Fig. 4, it can be noted that uniform data form is of one type, while the remaining 4 combination corresponds with multiple diversified data forms. Apart from this, all the 5 features have been assessed with respect to their samples corresponding to bipolar disorder, and a normal state has been carried out in both standalone form as well as in multiple possible combinations with each other. The model considers 45 final feature set after preprocessing and aggregation that represents integrated multimodal attributes utilized for training the model. It should be noted that 45 also represents dimensionality of the model.

### Rating estimation framework

The outcome of the prior system module is an aggregated feature which were adopted for training the forecasting framework in order to analyse ratings based on global standards^50,51^. The aggregated features were trained using a deep neural network that is capable of processing high-dimensional data, followed by learning complex world problems. The intricate patterns, along with all possible interactions of multi-modal features adopted in the proposed model, can be learned effectively by a deep neural network.

### Building a predictive model

Before constructing a predictive model, the prior step is towards developing a framework archive^52^. The proposed system constructs a framework archive and a hybrid ML model in order to append the outcomes for optimal decision making. According to this process of framework archive, the first part of the operation is associated with handling missing information. When a specific form of information (could be in the form of an attribute or feature) is found missing for a stated instance, the proposed model doesn’t discard the entire instance, nor does it do try to impute the data that is missing. Instead, pro-posed system selects the model that has not recorded to undergo any form of training operation using specifically that data recorded to be missing. Hence, better prediction integrity can be accomplished by this approach, that narrow down the selection of only those models that are actually capable of handling specific situations. Once the appropriate model is selected, the system integrates the predictive outcome using a hybrid learning model to achieve more predictive accuracy. Under this scheme, different types of ML models have been used for training, considering similar tasks but variable logical conditions. The predictive outcome of the proposed model is appended to arrive at the outcome. The overall operation towards the model selection under missing modalities unlike some recent schemes^53^ are as follows: (i) The modalities of available features are identified towards instances of test, (ii) The training models are selected without leaving aside any modalities, (iii) The selected models are used for yielding the predictions, (iv) Weighted averaging is carried out for aggregating the prediction, (v) The risk score of BD is finally yielded as an outcome. Public dataset is used for pretraining these models, while they are utilised as feature extractors, and these models are not subjected to training, considering the end-to-end dataset of a participant.

- *For Text Modality*: Sentiment embeddings are carried out using a pretrained BERT encoder with uncased base and 12 layers.
- *For Speech Modality*: This form of modality is processed using a semi-CNN architecture with 2 dense layers and 3 convolution layers.
- *For Video Modality*: This is processed using an architecture developed from the fusion of CNN and RNN, considering 3 blocks of CNN, while 64 units of LSTM are used.

### Framework archive strategy for missing modality handling

A framework archive strategy is adopted by the proposed IDMBD for addressing impartial multimodal input without depending upon classical imputation methods. The key objective of adopted strategy is towards retaining predictive integrity along with reduction of impact of artificially reconstructed information. The proposed model do not substitute any unavailable modalities with any form of estimated values and it retains aggregation of pretrained predictive models where combination of all available modalities are mapped with each model.

While obtaining inference of the model, the system initially looks for available modalities from the participant profile. The system search for the framework archive towards choosing those model that were trained using similar subset of available modalities. The computation of weighted aggregation towards predictive scores are carried out by the system upon findings presence of multiple eligible modes. Therefore, it can be stated that compatible models are necessary for performing prediction and the system do not force the reconstruction of complete modalities. The sequence of the archiving strategy of the framework is as follows:

1. The multimodal participating features are acquired from MCD.
2. The system attempts identifying any missing or any unavailable modalities.
3. The system detects archived models that are trained without any form of dependency towards missing modalities.
4. The prediction is performed using chosen compatible model.
5. The weighted aggregation is performed toward predictive scores.
6. Finally, the system generates estimation scores of BD risk.

Different from classical techniques of handling missing data (e.g. masking-based multimodal fusion, feature interpolation, mean imputation), the proposed model resists towards inducing ant form of synthetic data into behavioral data. This is quite essential consideration as emotional and behavioral modalities linked with BD could showcases non-linear dependencies and increased variability. Hence, signature of behaviour can be easily distorted by the standard imputation that can minimise the reliability of predictive performance. In contrast to approaches towards end-to-end multimodal fusion that demands comprehensive modalities all at same time during inference, the presented framework archive strategy that yields enhanced flexibility towards deployment. This is highly favourable for scenarios where sensing conditions are incomplete in mobile-centric ecosystem. The practical usability is also enhanced while computational overhead linked to reconstruction is minimised.

## Results and discussion

The proposed IDMBD framework is meant to collect various types of data using MCD, which, when fed to an adopted ML model, leads to the generation of a predictive outcome. Almost all the iOS and Android-based MCDs of any brand can be used for this purpose. For the current experiment, the extraction of data is carried out using an iPhone 15 with a wider range of fitness applications available from the Apple Store. The data is collected for 70 participants (35 participants have reported prior episodes of BD, while 35 participants belong to the normal group) over a period of 90 days. The aggregated data is used for training purposes. Once the training is accomplished, the framework of IDMBD is tested with various ranges of queried data captured from the same devices as well as from heterogeneous devices. The analysis is carried out to find the performance of IDMBD on various parameters.

### Dataset description and ethical considerations

The assessment of the proposed study model has been carried out using an experimental evaluation process where 70 voluntary participants were involved in collecting a longitudinal dataset. The dataset has been collected by monitoring the subjects for 90 consecutive days with the aid of mobile computing devices. There are 35 participants present within the cohort study with previous reporting of BD, while another 35 participants do not have any significant medical history related to mental illness.

- **Participant demographics**: The age group of the participants is 18–45 years, i.e., 28.6 years is the average age of the participants. The subjects involve both male and female gender with each subject holding a different form of educational and occupational background. Such participants were intentionally selected to ensure enough variability in behavioural patterns and lifestyle. Each subjects are regular user of their own smartphone and is found to be well aware of all possible applications on mobile computing devices.
- **Selection Criteria**: The inclusion criteria involves (i) age of each participants should be equal to or more than 18 years, (ii) they should be regular user of either android or iOS-based smartphone in their daily lives, (iii) the participants should be self-motivated to furnish lifestyle input and self-reported mood, (iv) for participants with BD, they should have been confirmed by qualified and certified physician while for participants without BD, there should be zero history of any mental illness. The exclusion criteria involve (i) any missing data of more than 30% of the observation window, (ii) subjects who don’t participate fully in the monitoring period, (iii) any subjects found/reported to have substance abuse, and (iv) critical neurological disorder
- **Ethics approval and informed consent**: The proposed study has complied with the ethical standards towards collecting data related to mental health. Informed consent was provided before registration to all the participants who joined voluntarily. All the data were kept confidential and anonymised before performing data analysis, and they were used only for research purposes. No information related to the personal identity of the participants was digitally stored in any place of experimental dataset preparation. As the proposed model functions adopting multimodal behavioral information obtained from devices of mobile computing, there is a practical consideration of security and data privacy towards practical environment usage. The participant information is anonymized before the analysis while access is restricted to processed dataset only to be used in research work. At the same time, it is much likely that behavioural information associated with contents of hybrid media, emotional indicators, and location traces could still induce concerns related to privacy sensitiveness in case of large-scale deployment. Hence, the practical deployment of proposed model will demand transparent user-consent policy, management of access controls, encrypted storage mechanism, protocols of secure communication that are complaint of regulations linked with healthcare data protection. Apart from this, the participant should possess the capability towards selective control of modality sharing as well as revoking consent whenever it is demanded. The learning strategies of privacy preservation will be the subjected of future work that will further involve blockchain-assisted management of data security and federated learning for further optimization. Such idea is also anticipated to offer security and trustworthiness towards screening any form of mental health.

It should be noted that all clinical labels used in dataset management for the proposed study associated with the group of BD are based on confirmation of a previous diagnosis of the same disorder by a certified psychiatrist. Labelling is also carried out considering self-reporting of the subjects, while there is no reported history of mental illness from the control participants. The proposed system uses the Kaggle dataset only for pretraining of feature extraction modules, while the collected participants’ dataset is used for performing predictive analysis and accomplishing reported outcomes. The study ensures the usage of demographic variables in the form of covariates for controlling any possibilities of bias.

### Clinical monitoring protocol

The proposed study has designed the monitoring protocol in order to simulate a practical form of longitudinal workflow screening that is appropriate towards monitoring mental health, considering mobile computing devices. There are various stages of this monitoring protocol that are briefed as follows:

- **Registration Stage**: All the baseline questionnaires were designed from standard questionnaires complying with the Altman Mania Scale and DAS-21. They have been filled out by participants in the form of an application that was installed on their phone.
- **Daily Monitoring Stage**: The participants are required to make an entry for self-reported mood once per day. The information related to sleep data, as well as the position of subjects were passively collected, followed by a hybrid-media (text/speech/video) collection weekly.
- **Periodic Assessment**: The features were aggregated weekly along with scores related to an intermediate risk. The system also checks the consistency and completeness of data once a month.
- **Final Evaluation**: All the features were collected for 90 days and then subjected towards training and testing for analysing the predictive models.

Adherence to these monitoring protocols assists in maintaining consistency and reproducibility towards participants.

### Model training and validation strategy

The study model has used participant-level separation to resist any possibility of data leakage. The data that were collected from the participants were involved only in training or the testing phase, but they were not used in both at the same time. This consideration attempts to ensure a higher degree of generalisation over different subjects, and not only towards retaining patterns specific to the subject. Owing to the adoption of longitudinal collection of data, the temporal sequences of the data are fused to generate features specific to the participant level before training the model. This task resists the model for any form of temporal leakage over both the training and testing datasets. The training is carried out considering splits of 80% of training data and 20% of testing data for predictive models, while it also retains class balance. The system also performs a 5-fold cross-validation in order to minimise the variance and thereby control overfitting possibilities. The outcome of validation is found to be corresponding to the mean across folds. An iteration of 20 independent runs was considered for performing training, considering an arbitrary initialisation, while bootstrapping is used for computing confidence and variance.

The scripting and implementation of the study model have been carried out using Python, while essential package libraries of TensorFlow and Scikit-Learn have been used with 63-bit windows machine with NVIDIA GPU. The CNN architecture consists of 3 × 3 kernel sizes with 3 convolution layers, ReLU activation layer, Max-pooling layers, dense layers (:128◊64), and Softmax layer. The LSTM architecture consists of 2 LSTM layers, where the first layer is of 128 units and the second layer is of 64 units. The dropout rate of 0.3 has been considered here. The hyperparameters used for the experiments are as follows: The analysis uses Adam optimiser with 0.001 learning rate, 50 epochs, and multiple batch sizes (32-64-128). A dropout rate of 0.2 is considered, while the early stopping criterion is formed depending upon the validation loss. The selection of hyperparameters are carried out using prior domain knowledge and empirical evaluation in order to accomplish an equilibrium between computational efficiency and model performance. The proposed study didn’t use formal strategy of grid search; instead, it has adopted a limited manual tuning method for minimizing computational overhead.

The proposed system implements a sensitivity analysis towards investigating the impact of chosen hyperparameters on predictive stability. A batch of varying size (32, 64, 128) has been considered in the study along with consideration of dropout rate (0.2–0.3). The assessment also chooses a learning rate which are around configuration of selected baseline. It has been noted that surplus sizes of smaller batches to maximize the instability of training while marginal minimization of generalized performance is noted from usage of batches of larger sizes. In same way, the overfitting possibility increases with minimized rates of dropouts metric while it also minimizes the predictive accuracy. A balanced trade-off among resource-aware deployment suitability, predictive stability, and computational efficiency has been witnessed upon selection of chosen configuration of hyperparameters on mobile-centric ecosystem. As the proposed model focuses on feasibility of lightweight implementation, the system intentionally avoid using grid-search optimization in order to control iterative training overhead or computational complexity. The study has used training data for performing hyperparameter tuning within framework of cross-validation for resisting any form of biasness and ensuring highly robust evaluation of model.

- **Reproducibility and Implementation Details**: In order to ensure reproducibility of the system model, it is essential to realize the implementation and architectural details in discrete mode. The rating estimation is carried out by DNN which consist of 5 feature groups within its input layers while its hidden layers consists of two fully connected layers of 128 and 64 units. The output layer of DNN consists of SoftMax activation needed for binary classification. Further, hidden layer is also characterized with implementation of Rectified Linear Unit (ReLU) which system applies batch normalization upon stabilized training of each hidden layers. The system uses pretrained model for feature extraction of hybrid media. Text embeddings were carried out by BERT (uncased, 12 layers) while speech processing is carried out using semi-CNN architecture (with 2 dense layers and 3 convolution layers). The video analysis is carried out using hybrid CNN-RNN model (with 3 blocks of CNN and 64 units of LSTM). Adam optimizer is used for training considering 0.001 as learning rate, 32 as batch size, and epochs of 64-128-50. The system uses 0.2 and 0.3 as range of dropout rates necessary for accomplishing regularization while validation loss is used for early stopping criterion. The system also applies a unique technique for handling missing modality. It uses framework archive strategy which makes a selection of specific models during inference that were without missing modality. Adherence of this strategy ensures of better consistency as well as resists any form of imputation biases while making prediction. Instances with inclusion of 30% missing data or more is considered for removal under preprocessing steps. Further, feature values were normalized while temporal signals were aggregated and scaling of mean and variance were used for standardizing feature. The complete logic has been scripted in Python where scikit-learn and TensorFlow libraries have been used considering 64 bit windows system enabled with GPU. All this information ensures of better reproducibility as well as enhanced scope of validation in further direction of this work.
- **Model robustness vs. sample size**: The proposed system implementation consists of multiple safeguards for overcoming possibility of overfitting despite the usage of relatively limited size of cohort (i.e., *n* = 70) for ensuring robustness of IDMDB model. For this purpose, a strict strategy for separation of participants was adopted which will offer an assurance that information from any set of unit subject can be used either for testing or for training but not for both at same time. This strategy assists towards better generalization capability and also resists any possibility of subject-centric bias. Further, 5-fold cross-validation has been adopted for assessing the model training where class balance is preserved by each fold. The performance parameters used for this purpose are mean and variance across fold while an arbitrary initialization is carried out for 20 independent runs of an experiment. The computed variance in accuracy over the experimental runs is found within a tight margin of ± 1.79% stating that the proposed model is quite stable. Apart from this, overfitting and model complexity are controlled by adopting batch normalization, early stopping based on validation loss, and dropout rates (0.2–0.3) as regularization strategy. The estimation of reinforce statistical reliability and confidence intervals were carried out by bootstrapping 1000 samples. With accomplishment of increased accuracy, the system interprets this score in the context of multimodal enriched features and controlled condition of experiment. Hence, the model can be stated as robust tool for early diagnosis rather than considering it as a definitive system for clinical diagnostics.

In order to resist any feasibility of data leakage, the proposed system has carried out operations only after the split of train-test data of participant levels. The dataset was initially classified into training set and testing set while each individual sets have been independently used for subsequent computation (extraction of feature, preprocessing, fusion). This phenomenon offers an assurance that training process is never influenced by any information within test data which retains the evaluation validation to higher degree. The next section presents discussion of assessment strategy adopted for experiment.

### Assessment strategy

The proposed system considers various standard parameters in order to assess the performance, viz., accuracy, precision, recall (sensitivity), specificity, F1-score, ROC-AUC, and confusion matrix analysis. Probability reliability curves have been used for calibrating the prediction.

The core module of IDMBD uses DNN with an input layer with five input arguments (based on computed features), two hidden layers, and one output layer. Rectified Linear Units have been used as an activation function for hidden layers, while SoftMax is used for the output layer for classification. Adam optimiser is used as an optimiser, while 0.001 is used as a learning rate decay factor. Cross-entropy has been used as a loss function towards binary classification, while 0.2 is considered a dropout rate as a regularisation technique in order to resist overfitting. The proposed IDMBD uses 32, 64, and 128 as batch sizes for forward passes, while 50 is considered the number of epochs. Further, a batch normalisation layer is added to normalise the input associated with each layer. Further, the analysis is carried out by three different combinations as follows:

- **Combination-1 (IDMBD1)**: IDMBD is integrated with CNN and LSTM. CNN is known for its superior predictive accuracy, while LSTM is known for its capabilities towards temporal sequences of information. So, it is hypothesised that IDMBD1 could offer significantly superior performance, especially with respect to accuracy.
- **Combination-2(IDMBD2)**: IDMBD is integrated with RF and DT. RF and DT are the simplest ML algorithms with efficient predictive performance in cost effective manner. As the core architecture is anticipated to be presented in the form of an underlying MCD, this combination is meant to testify to the resource-effectiveness along with predictive accuracy.
- **Combination-3(IDMBD3)**: IDMBD is integrated with RF, DT, and SVM. There are various reported literatures where SVM has played a contributory role in the diagnosis of various mental disorders. Hence, this combination hypothesises that if SVM is integrated with cost-effective ML algorithm e.g., RF and DT, there are possibilities of much better predictive performance by IDMBD.

All the above-mentioned triple sets of combination was used to evaluate the model’s effectiveness to explore its scope and efficiency towards early diagnosis of BD. The assessment outcome has been evaluated with respect to four performance metrics, viz., (i) Predictive Accuracy is computed as the number of appropriate predictions made by the IDMBD divided by the total number of predictions. For simplification, it is represented in the form of a percentile for a better inference system. (ii) Training Time is calculated by the overall duration demanded for training the IDMBD model. (iii) Response Time is calculated as the duration consumed by the IDMBD model to carry out any prediction on untrained data. (iv) Memory Saturation is calculated using the psutil library and refers to consumed RAM while training and testing for IDMBD is carried out. All the above performance metric is applied while performing comparative analysis of the proposed IDMBD with various standalone existing ML models, viz. RF, SVM, NB, LoR, LiR, KNN, and DT. In order to offer statistical reliability, the proposed system entails 95% of confidence interval (CI) computation, and this is obtained when 1000 resamples were subjected to bootstrapping. The mean performance is represented by the reported values of accuracy over iterative experiments carried out on training-testing splits of data. The statistical transparency is ensured by computing confidence interval adopting 1000 samples using non-parametric bootstrapping method. The system recalculates all the performance metrics for each resample followed by obtaining 95% of confidence interval acquired from empirical distribution adopting percentile evaluation. Further, the study also computes statistical significance between baseline and IDMBD model adopted paired t-test over folds of cross-validation. The assessment has considered *p* significance value less than 0.05 for representing statistical significance score. It was noted that reported *p* significance value towards performance metric satisfies this criterion in order to confirm that proposed system offers reliable outcomes for its model implementation.

### Accomplished results

**Fig. 5:**
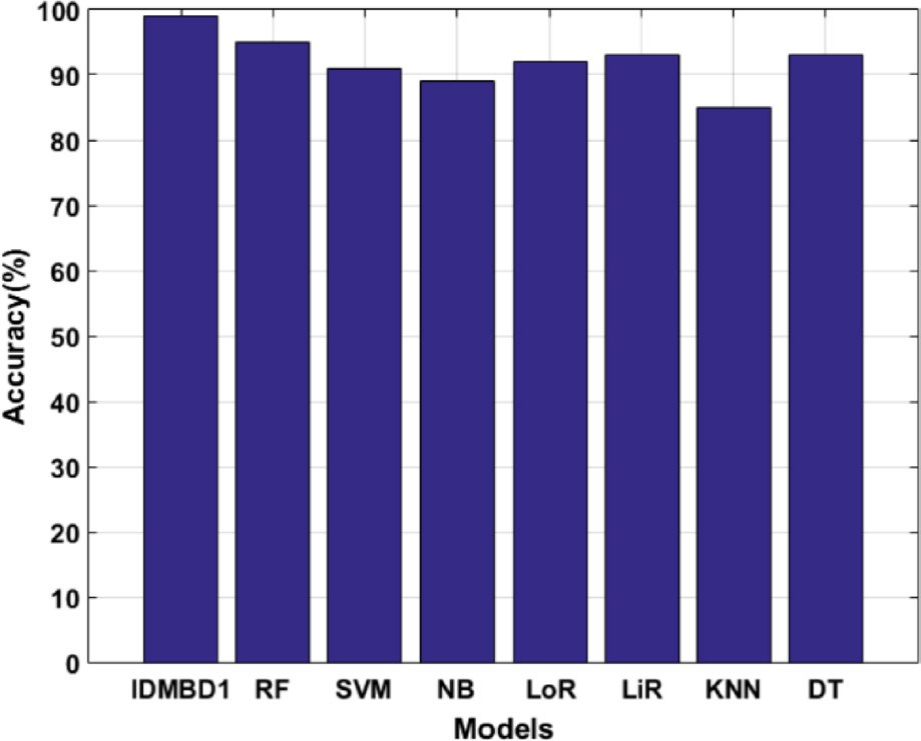
Comparative Analysis of Accuracy for Combination-1.

**Fig. 6:**
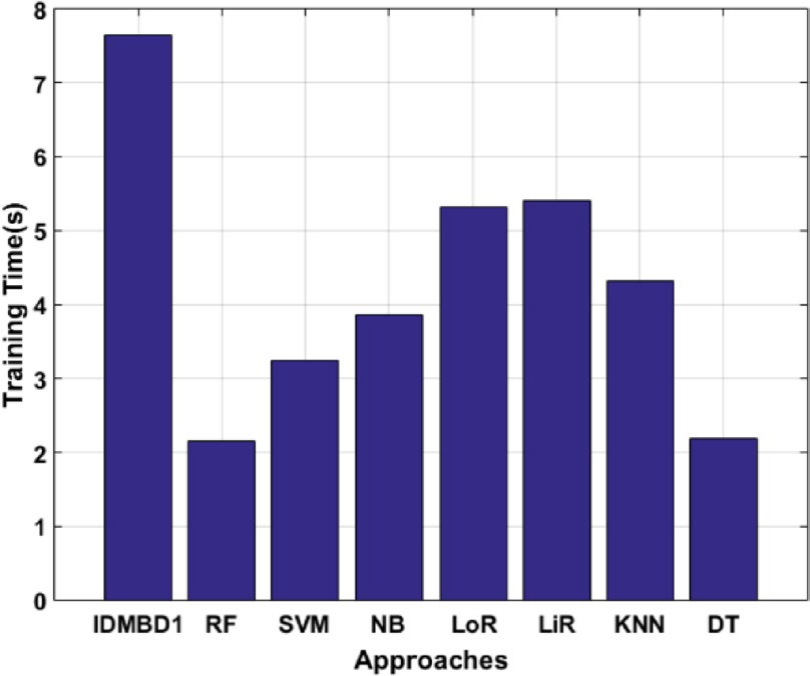
Comparative Analysis of Training Time for Combination-1.

**Fig. 7:**
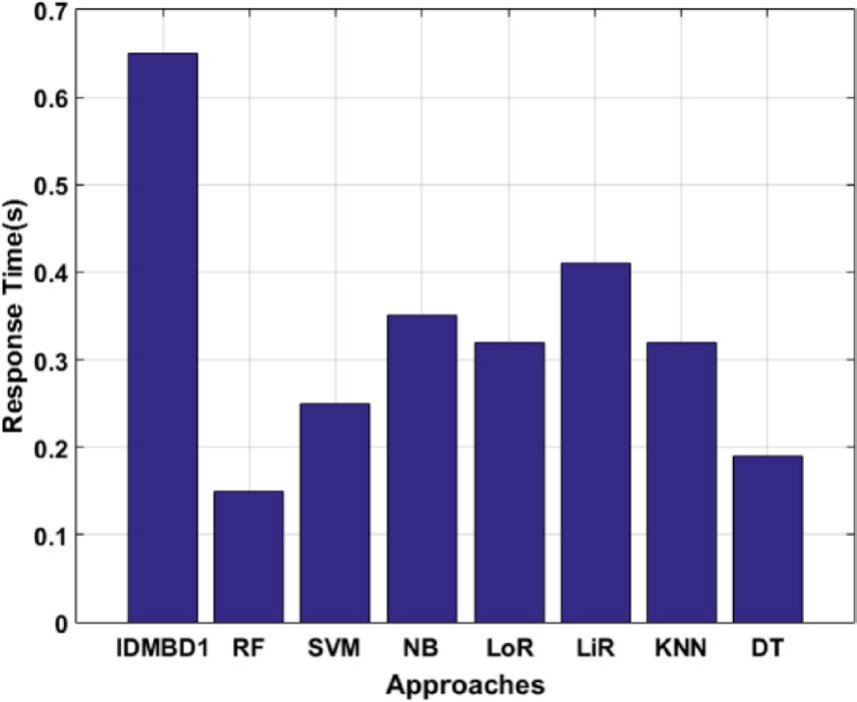
Comparative Analysis of Response Time for Combination-1.

**Fig. 8:**
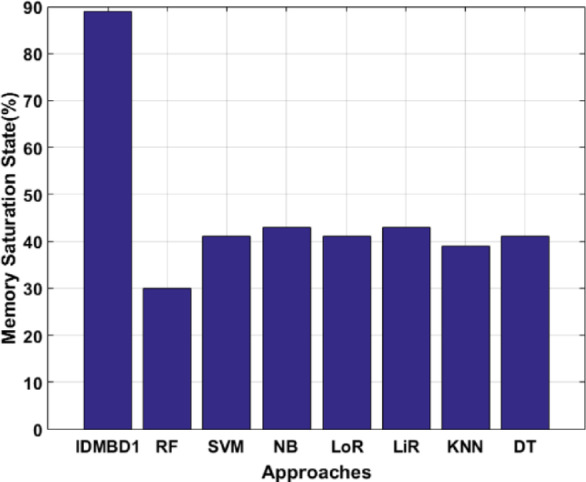
Comparative Analysis of Memory Saturation State for Combination-1.

**Fig. 9:**
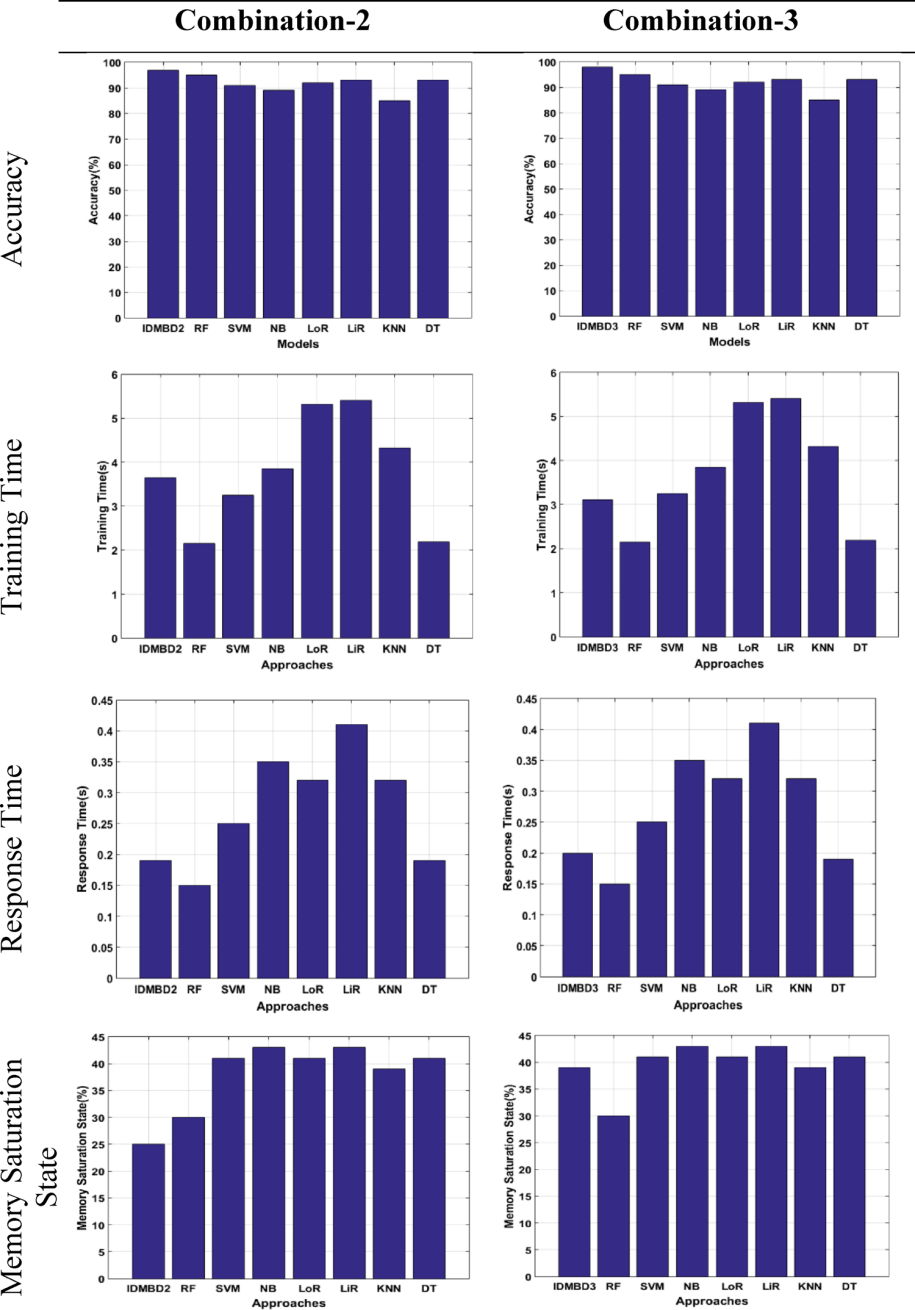
Parallel Assessment of 2nd and 3rd Combination Model.

The numerical score of the accomplished result for Combination-1 is shown in Table 2. The outcome evidently shows that IDMBD1 has a superior predictive accuracy score, which is mainly due to the involvement of CNN in contrast to other ML models. However, IDMBD1 also witnesses the highest training, delayed response time, and increased memory saturation score in contrast to existing ML models. Figures 5, 6, 7, 8 and 9 offers simplified graphical insights towards easier inference.

**Table 2:** Comparative Analysis using Combination 1.

Approach	Accuracy (%)	Training Time (s)	Response Time (s)	Memory Saturation (%)
IDMBD1	99	7.65	0.65	89
RF	95	2.15	0.15	30
SVM	91	3.25	0.25	41
NB	89	3.85	0.35	43
LoR	92	5.32	0.32	41
LiR	93	5.41	0.41	43
KNN	85	4.32	0.32	39
DT	93	2.19	0.19	41

The graphical representation showcased in Figs. 5, 6, 7 and 8 showcases the simplified interpretation of trade-off between computational efficiency and predictive performance over multiple learning strategies implemented within the proposed model.

A closer insight to graphical outcomes exhibited in Figs. 5, 6, 7 and 8 shows that CNN-LSTM has excel higher predictive accuracy but at the cost of extensive resources. This can be realised by observing IDMBD1 with increased training time that normally uses backpropagation while training a deep LSTM and CNN model. Further, early diagnosis of BD demands IDMBD1 to be speedily. Unfortunately, CNN-LSTM demands the inclusion of a large number of parameters, while more computational overhead can be generated due to convolution layers integrated with sequential processing. Further, inclusion of data buffering, storage of sequential data, and a large number of parameters are prime attributes resulting in higher memory consumption. Buffering a large amount of data in resource-constrained MCDs for supporting real-time incoming data/readings is further quite challenging. A closer look into training time (Fig. 6) and memory saturation time (Fig. 8) will show that IDMBD1 is a highly computationally intensive model. Apart from this, it is known that the core model of IDMBD performs local processing as well as global processing; however, this local processing is carried out within the MCD itself, which significantly affects the memory constraint and processing time, especially in local processing. However, global processing relaxes this constraint by offloading the complete computation to the distributed servers in the cloud. Hence, the conclusive remark for IDMBD1 is that when IDMBD is integrated with CNN and LSTM, predictive accuracy is the best part of the accomplishment; however, there is a sharp degradation of other performance parameters. Out of existing ML models, RF and DT are the optimal performers in almost all the performance metrics except accuracy when compared with the CNN-LSTM model. Hence, IDMBD1 shows its good result by exhibiting 8% of increased accuracy; However, IDMBD1 exhibits its degraded performance by showing 38% of increased training time, 26% of increased response time, and 49% of increased memory saturation state in contrast to other models.

**Table 3:** Comparative Analysis using Combination 2.

Approach	Accuracy (%)	Training time (s)	Response time (s)	Memory saturation (%)
IDMBD2	97	7.65	0.19	25
RF	95	2.15	0.15	30
SVM	91	3.25	0.25	41
NB	89	3.85	0.35	43
LoR	92	5.32	0.32	41
LiR	93	5.41	0.41	43
KNN	85	4.32	0.32	39
DT	93	2.19	0.19	41

**Table 4:** Comparative Analysis using Combination 3.

Approach	Accuracy (%)	Training time (s)	Response time (s)	Memory saturation (%)
IDMBD3	98	3.65	0.20	39
RF	95	2.15	0.15	30
SVM	91	3.25	0.25	41
NB	89	3.85	0.35	43
LoR	92	5.32	0.32	41
LiR	93	5.41	0.41	43
KNN	85	4.32	0.32	39
DT	93	2.19	0.19	41

An interesting part of the previous observation is that RF and DT have evolved to be optimal performers, and hence a trial was carried out by combining the proposed IDMBD with RF and DT (as shown in Table 3). Further, a closer look at the sequences of numerical scores in Table 3, it can also be realised that perhaps SVM stands another better chance for optimising model performance. Hence, another trial is carried out where the proposed IDMBD is integrated with SVM, along with retaining RF and DT models (Table 4).

For a better understanding of this comparison, we offer graphical insights in Fig. 9, where it can be realised that IDMBD2 (IDMBD with RF and DT) is exhibiting better performance in the majority of the times in contrast to IDMBD3 (IDMBD with RF, DT, and SVM). It is concluded that SVM is undeniably a robust ML model in its standalone form, and it will not be a wise decision to increase the computational complexity by adding SVM on top of the RF and DT integrated IDMBD model. Hence, this outcome concludes that RF and DT are the best performing algorithms when working with IDMBD, while SVM also stands a good chance to be integrated with IDMBD. However, comparisons do show a significant distinction in both the outcome exhibits to arrive at the inference that other ML algorithms are equally good if the problem investigated could be related to only analysis (or prediction) of BD; however, when it comes to early stage detection, one demands a lightweight system, can run sustainably, with consistent scores of predictive accuracies that can be witnessed with RF and DT. Further, additional analysis outcome is shown in Table 5.

**Table 5:** Additional Analysis Performance of IDMBD and Baseline Models.

Model	Specificity (%)	Sensitivity (%)	AUC score
DT	92	93	0.93
NB	88	89	0.90
SVM	90	91	0.92
RF	95	94	0.95
IDMBD3 (RF + DT+SVM)	96	97	0.98
IDMBD2 (RF + DT)	96	96	0.97
IDMBD1 (CNN+LSTM)	97	98	0.99

The Table 5 above shows three different variants of IDMBD with combination of multiple models compared with standalone baseline models. The outcome shows that ROC-AUC scores for all variants of IDMBD exhibits potential discriminative power while higher AUC is accomplished by IDMBD1 while lower computational cost is exhibit by IDMBD2 variant. It should that IDMBD2 is suitable for resource-constraint environment implementation. IDMBD also exhibits highly balanced performance for sensitivity and specificity in detection of BD and non-BD use-cases. Effective early detection is facilitated by higher score of sensitivity while reduction in false positives are exhibited in higher value of specificity. This eventually showcase notable performance gains for all variants of IDMBD in contrast to baselines.

**Fig. 10:**
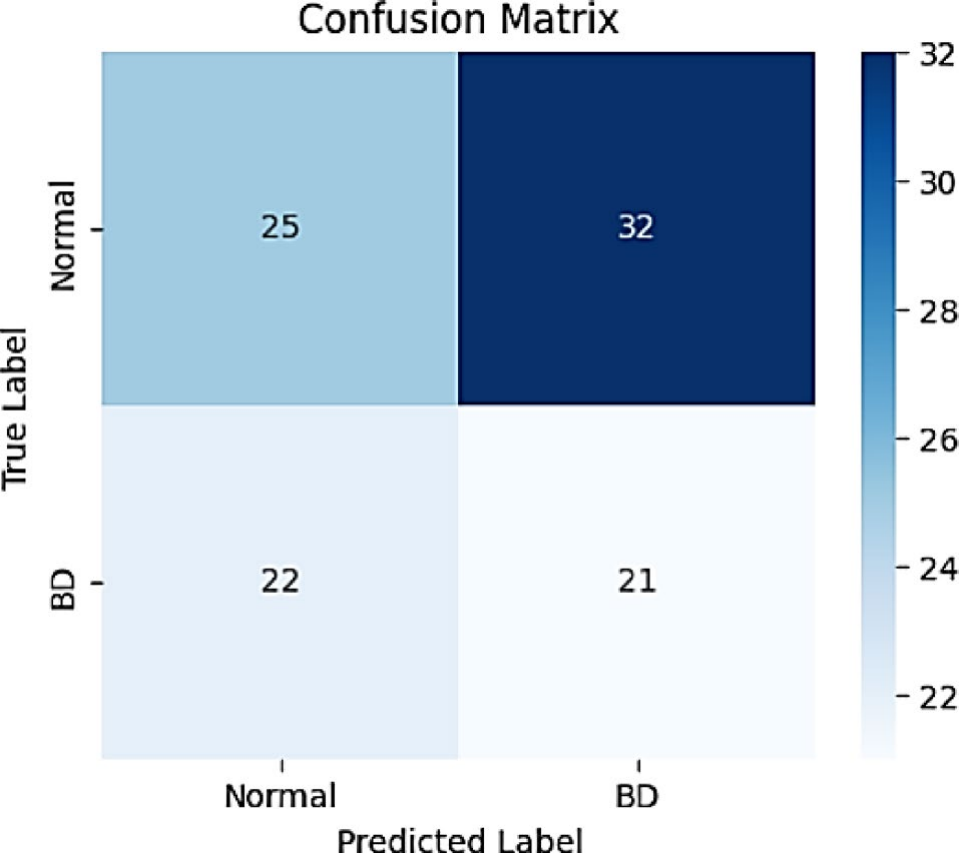
Confusion Matrix.

Figure 10 showcases the confusion matrix for exhibiting the illustrative understanding out classification result. The diagram exhibited in Fig. 10 towards confusion matrix shows the capability of balanced classification linked with proposed IDMBD towards both BD as well as non-BD participant classes with minimized rates of misinterpretation. A closer look into the confusion matrix will show that there are reduced number of false negatives which is quite essential for early detection of BD where any form of missed diagnosis is also quite important to be considered towards understanding clinical consequences. Apart from this, the system also exhibits low rate of false positives representing the capability of IDMBD towards resisting unwanted outcomes. Thereby, the confusion matrix showcases proposed IDMBD model as reliable screening tool.

**Fig. 11:**
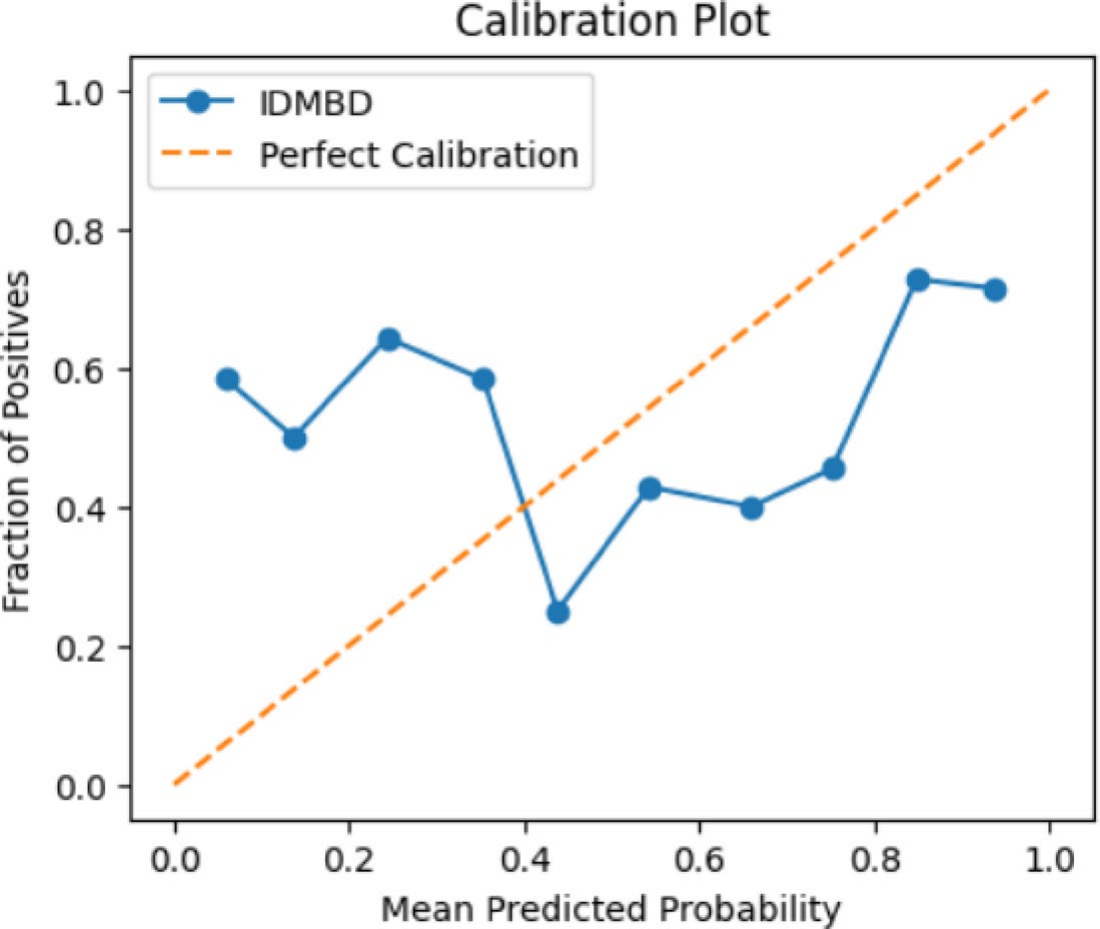
Calibration Plot.

Figure 11 showcases a calibration plot that exhibits a proper agreement is existing between observed values and predicted values of probabilities. The calibration behaviour showcased in Fig. 11 is found to be supportive of trustworthiness linked with probabilistic predictive outcome yielded by IDMBD under multimodal condition of behavioral assessment. A closer alignment of IDMBD model can be witnessed here with ideal line of calibration which shows reliable estimation of probability towards assessment of clinical risk. The outcome trend also suggest that IDMBD models not only yields better classification scores but also generates highly trustworthy scores too that is much necessary for clinicians for understanding the practical level of associated risk more effectively. Such form of calibration is quite necessary for an effective decision-support system where under or over confident scores of prediction could yield an irrelevant intervention leading to misjudged diagnosis.

The study has carried out a paired *t*-test for assessing the statistical reliability by comparing the proposed model with baseline methods while performing cross-validation folds. The outcome finds both F1-Score and accuracy to be statistically significant at *p* < 0.05. Further, an extensive analysis has exhibited Random Forest to be performing better in every performance metric, especially in terms of robustness, while interpretability is well achieved by the decision tree. Although higher version of competitive performance is witnessed by deep models, such optimal outcomes are yielded at the cost of reliance on a massive quantity of data.

### Ablation study

The proposed model has been assessed by performing an ablation study, which has attempted to evaluate the contribution of the standalone feature modality involved in the IDMBD framework. The study considers various combinations of feature modality in order to understand its significance. The following are the considered feature modalities involved in the ablation study:

- **Full IDMBD (with complete feature)**: This consists of a complete system model with sleep data as well as information related to hybrid media, emotion, personal assessment, positional data, etc. This combination is used to capture the complete multimodal representation for early detection of BD.
- **Without Positional Information**: The significance of positional data is towards tracking mobility variation and daily movement patterns that are capable of reflecting behavioural changes connected with mood instability.
- **Without Personal Assessment Measure**: Inclusion of self-assessment scales offers a clear inference towards the manic symptoms, anxiety, or depression. This measure is highly essential for the early identification of BD.
- **Without Emotional Well-being**: This metric is used for monitoring the emotional state continuously for understanding the fluctuation of mood over time. It facilitates fine-grained signals of behaviour for forecasting.
- **Without Hybrid Media**: The extraction of subtle affective cues is facilitated by hybrid media like video, speech, text, etc. This makes the content a highly critical format towards detection of early indicators of BD.
- **Without Sleep Cycle**: There is a potential correlation between sleep patterns and the onset of BD as well as progression. Hence, inclusion of information related to the sleep cycle essentially enhances the predictive accuracy.
- **Without Hybrid Media & Emotional Well-being**: This combination exhibits the significance of influenced signals as eliminating both potentially minimises the performance of model prediction and hence exhibits their role.

Adoption of all these combinations in an ablation study facilitates quantifying the relative emphasis towards each feature modality as well as confirming the robustness associated with the complete proposed model. Similar participant-level splits for both training and testing data were used for performing the experiments, while a 5-fold cross-validation strategy was used. Table 6 showcases the numerical outcomes accomplished from the study.

**Table 6:** Ablation Study Results of IDMBD Framework.

Feature modalities included	Predictive accuracy (%)
Full IDMBD (All Features)	97
IDMBD without Positional Information	92
IDMBD without Personal Assessment Measure	90
IDMBD without Emotional Well-being	91
IDMBD without Hybrid Media	89
IDMBD without Sleep Cycle	93
IDMBD without Hybrid Media & Emotional Well-being	87

The results in Table 6 showcase that the complete IDMBD framework excels in every perspective in contrast to its counter models with a reduced version. It should be noted that the model has conducted a preliminary correlation analysis for ensuring minimization of redundant features. This yields to minimization of multicollinearity to higher degree as well as enhance the stability of IDMBD model. It directly states that inclusion of all the modalities is highly essential for stating the efficient predictive performance of IDMBD. Out of different standalone modalities, hybrid media is found to exhibit the strongest influence on system performance, which is further followed up quite closely by emotional well-being and personal assessment measures. The ablation study also finds that sleep cycle and positional information have quite a reduced influence as a standalone feature modality, while it contributes towards improving predictive accuracy when integrated with different modalities.

The outcome of the ablation study shows the positive strength of IDMBD. The model fuses physiological signals with diverse behavioural information for accomplishing maximised predictive reliability in contrast to any partial or single feature set. The outcome also offers a rationale behind the selection of hybrid DNN, RF, and DT towards analyzing heterogeneous input data. Apart from this, the proposed study also showcases that the predictive performance of IDMBD doesn’t depend disproportionately on any form of single modality. These facts are in support of higher robustness and generalizability of IDMBD towards detecting the BD in the early stage.

Table 7 showcases the comparison being carried out between proposed model and state-of-the-art methods presented in Sect.  2 of related work. It has been noted that existing methods reported in literature usually make use of single modality or adopt clinically constraint environment with computationally intensive DL models. On the contrary, proposed IDMBD collaborates 5 different behavioral modalities on the top of mobile-centric framework along with equal emphasis towards resource-aware prediction. This comparison also showcases that proposed model accomplishes robustness and highly balanced predictive performance even in presence of missing modalities via its framework archive strategy. It also minimizes the dependencies towards deployment in contrast to previous DL or multimodal approaches. The practical suitability of proposed IDMBD is further strengthened via this comparison towards early stage assessment of BD even in presence of restricted resource environment. Table 7 shows A_1_: Contribution, A_2_: Reported Accuracy (%), A_3_: Modality Count, A_4_: Computational Dependency, A_5_: Observed Limitation, and A_6_: Improvement Achieved by IDMBD.

**Table 7:** Comparison with current state-of-the-art models.

Refs	A_1_	A_2_	A_3_	A_4_	A_5_	A_6_
^20^	hypomania feature selection using RF	~ 90	1	Low	Questionnaire-centric analysis	IDMBD integrates heterogeneous multimodal behavioural profiling
^21^	BD classification using ML	~ 93	1	High	Requires specialised MRI infrastructure	IDMBD enables mobile-centric lightweight deployment
^22^	Voice analytics for BD	~ 83	1	Medium	Restricted to speech modality	IDMBD combines speech, text, video, sleep, emotion, and positional data
^23^	Mental health prediction using SVM-MLP-RF	~ 92	2	Medium	General mental health prediction only	IDMBD specifically targets early-stage BD screening
^24^	Depression detection using SVM & neural network	~ 94	2	High	Focus limited to depression analytics	IDMBD investigates bipolar disorder behavioural irregularities
^25^	Multimodal MRI with ML	~ 95	2	High	Neuroimaging dependency and computational overhead	IDMBD avoids specialised sensing infrastructure
^26^	Abnormality classification with EEG & cognitive	~ 93	2	High	Requires EEG acquisition setup	IDMBD eliminates wearable and clinical sensing dependency
^27^	ML prediction (Neuroimaging & clinical)	~ 91	2	High	Resource-intensive framework	IDMBD emphasizes deployment feasibility
^28^	Depression detection using Motor activity	~ 87	1	Medium	Limited behavioural representation	IDMBD enriches profiling with diversified modalities
^29^	Logistic ridge regression with mRNA	~ 88	1	High	Requires biological/genetic testing	IDMBD avoids invasive laboratory analysis
^30^	BD Detection (Radial basis neural network)	~ 96	1	High	Increased architectural complexity	IDMBD balances performance with computational sustainability
^31^	Mental disorder prediction (ML, ensemble, LLM)	~ 93	2	High	Social-media dependency and privacy concerns	IDMBD operates on controlled voluntary behavioural sensing
^32^	Neuroimaging (Bayesian Regression)	~ 86	1	Medium	Limited mobile deployment feasibility	IDMBD explicitly supports MCD-based implementation
^33^	Mood forecasting	~ 85	1	Medium	Focused only on mood forecasting	IDMBD performs integrated BD risk screening
^34^	Facial micro-expression detection (ML)	~ 90	1	Medium	Single behavioural modality	IDMBD uses multimodal feature fusion
^35^	Biochemical biomarkers (DT)	~ 92	1	Medium	Biochemical testing dependency	IDMBD avoids pathological assessment requirements
^36^	Psychiatric differentiation (DT)	~ 88	1	Medium	Clinical dependency	IDMBD facilitates continuous behavioural monitoring
^37^	Signal discrimination (Speech)	~ 90	1	Medium	Limited modality diversity	IDMBD integrates hybrid media with behavioural sensing
^38^	Characterization (Emotional state)	~ 87	1	Low	Emotion-focused standalone analysis	IDMBD fuses emotional, sleep, and positional variability
^39^	Depressed mood detection	~ 93	1	Medium	Focus limited to depressive behaviour	IDMBD addresses BD-specific multimodal irregularities
^40^	DL with EEG data	~ 97	1	Very High	EEG acquisition and deep computational overhead	IDMBD achieves lightweight deployment feasibility
^41^	Mood detection (Attention CNN-LSTM)	~ 96	2	Very High	Resource-intensive deep architecture	IDMBD balances prediction with resource-awareness
^42^	Feature fusion (Hybrid CNN-BiLSTM)	~ 97	2	Very High	High computational complexity	IDMBD emphasizes sustainability for resource-constrained MCDs
IDMBD	BD risk screening with framework archive strategy (Multimodal mobile-centric)	97–99	5	Medium	Requires broader external clinical validation	Balanced multimodal profiling with lightweight deployment feasibility

The next section further offers more insight to inference of the outcome accomplished in the proposed study.

### Discussion

The superiority of the RF + DT hybrid configuration within IDMBD can also be attributed to its ability to balance bias-variance trade-off in the presence of heterogeneous multimodal features. While RF reduces variance through ensemble averaging, DT contributes to capturing localised decision structures, which is critical for modelling behavioural irregularities associated with early-stage BD. The accomplished result presented in the prior sub-section shows RF and DT to be the best performers while investigating early detection of BD. The prime reason for the increased accuracy of RF is due to its bagging technique, along with the adoption of multiple decision trees. RF, when combined with DT and core IDMBD, is found to be much less prone to overfitting. It is to be noted that feasibility of the remnant overfitting cannot be entirely mitigated which is mainly due to high-dimensional multimodal data with restricted size of cohort. The study has implemented multiple validation strategies as well as multiple regularization process. The complete model has been planned to be experimented with larger independent cohort in future to confirm the stability and long-term robustness of IDMBD. Also, it is interesting to find that when proposed IDMBD is integrated with RF and DT, its overall performance is much better compared to the standalone performance of RF and DT. It will eventually mean that DNN models used in IDMBD actually optimise the performance of RF and DT. Another justification for this outcome can also be attributed to the adoption of multimodal features that offer a comprehensive ground for network layers. This offers clearer filtering of the more dominant outcome compared to the less dominant outcomes in simpler steps.

When implemented with NB, it was noted that the training operation had simplified the calculation by assuming the independent nature of the 5 features. However, such heterogeneous forms of features are quite challenging to assume independently. It is because the features extracted from MCD can be highly correlated, which is not considered by NB model, resulting in decreased performance when encountered with complex forms of feature relationships. Further, LoR and LiR have also noted faster training operation; however, this faster performance consistently reduces when the size of incoming data increases. This potentially affects the training time and response time eventually, although LiR is quite storage efficient and demands minimal memory footprints. Another interesting part is that while using KNN, which doesn’t demand any form of training phases, it suffers from a high cost of computational operation, especially while performing prediction. KNN is also not recommended towards resource constraints MCDs as it will store all training information, saturating the complete memory. Hence, the following are the conclusive statements of accomplishment of the proposed study outcome:

- IDMBD offers reliable accuracy performance when integrated with RF, DT, and even SVM (to some extent). With a minimal cost towards training time and memory consumption, this combination offers optimal performance for early detection of BD.
- When it comes to computational speed, IDMBD can be integrated with only RF, which can make the module much lighter and faster in its operation (although its accuracy without DT can be negligibly affected).
- IDMBD offers potential control on overfitting and suitable usage of resources, which is mainly due to the overall core architecture itself. With supportability of processing and analysing various modes of features, IDMBD is capable of identifying exact trigger points (with respect to the feature) that lead to the onset of BD. It also means it is highly scalable when subjected to real-time environment.

The proposed model of IDMBD showcases effective predictive outcomes that could be interpreted in the context associated with controlled experimental settings and restricted size of cohort. Although overfitting problem has been sorted using different approaches (cross validation, bootstrapping, iterative experimentation, participant level separation), there is a still a possibility of depicting features specific to investigated cohort. This is the reason that overall findings of the current study should be considered as preliminary proof of implementation possibility and it should not be considered as definitive proof of large-scale implementation on clinical settings. The limitation of the study is absence of external validation adopting independent cohorts from different clinical environment and demographics while this limitations will be addressed in future line of implementation. The upcoming stage of implementation will also emphasize upon performing validation studies adopting large-scale multimodalities in order to evaluate the long-term clinical applicability, transferability, and robustness of IDMBD.

Hence, on this ground, it can be stated that IDMBD is proven with better predictive performance in contrast to various existing ML models. The prime limitation associated with the proposed study model is that it is carried out considering a relatively small size of the cohort (*n* = 70). As the size of the cohort is relatively small, careful interpretation is carried out throughout the manuscript. One important concern is that although the study has employed participant-wise splitting and cross-validation, it is necessary to consider larger as well as multi-center dataset for retaining maximum generalizability. Hence, it is essential to consider and emphasise external validation in future work direction towards adopting an independent dataset as well as collaborating with clinical assessment for assessing the robustness of the proposed model.

From the perspective of real-time deployment, the proposed framework of IDMBD is looked upon as a layer of assistive screening collaborated with current state of clinical operations. The indicators of multimodal behaviour is consistently aggregated by the system between estimated risk in early stage and scheduled consultation towards upcoming assessment. The healthcare professionals get immense benefit out of it by enhancing the screening efficiency, longitudinal screening of behavioral irregularities, and prioritizing the follow-up assessment towards vulnerable person. However, the model was not subjected to any clinical validation under real-world environment of psychiatric operations. Therefore, the final outcome obtained from proposed study should not be looked upon as standalone clinical diagnosis. Such shortcomings will be investigated in new direction in upcoming direction of future work. Irrespective of the fact that IDMBD offers promising performance gains exhibited in its result, its generalizability is still constrained by setting of data collection from single sources and limited size of considered cohort. The internal validity has been significantly enhanced by employing strategies of cross-validation and participation-level validation; however, it the model doesn’t offer a coverage for external validation adopting independent dataset from different clinical settings and demographics. Apart from this, the multimodal and behavioral patterns linked with bipolar disorder could eventually vary with respect to cultural context, geographical region, and population. Hence, a higher degree of caution is required while reporting the direct generalization performance over wider scope of population. Hence, future work could eventually emphasize on external validation adopting independent cohorts along with focus on multi-center collection of data on large scale. The applicability of the proposed model in practical world could be further strengthened by integrating validation with clinical workflow against longitudinal psychiatric assessment.

## Conclusion

A significant feasibility associated with the proposed IDMBD model has been noticed in the perspective of mobile-centric multimodal analytics, with an ideation of novel risk screening of BD in the early stage. Although the outcomes have exhibited a highly promising nature, the framework is potentially recommended to be assumed as a decision-support and research prototype. At no point should it be considered as a standalone tool for performing clinical diagnosis. At present, proposed IDMBD should be considered as a framework to offer research-centric decision support rather than any form of diagnostic system with clinical validation. The core contribution of the proposed model is as follows:

- IDMBD uses a universally accepted standard scale towards predicting the severity degree of BD among participants using profiling approaches. An MCD is used for acquiring all necessary features from the raw data, which can be done by any participant with minimal effort and no extra spending.
- IDMBD uses a framework archive for mitigating the issues pertaining to missing information. This framework archive consists of different ML models that are anticipated to work efficiently for multiple combinations.
- A different form of investigation strategy has been formulated, which not only assists in evaluating the current state of the model but also gives a fair insight into future probability. The current model uses DNN as a core part; however, it offers greater flexibility to a greater extent when integrated with RF and DT. This extensive analysis offers a wider scope for any researchers to further customise it according to their needs.
- The proposed system offers a reliable outcome, which can be stated on the basis of an extensive and diversified assessment environment with three different combinations of learning models.

The proposed study also demands more extensive validation, considering real-world deployed data and much larger clinical cohorts prior to actual clinical adoption. This is the prime limitation of the current work. Hence, future work could be carried out in this direction by implementing federated learning to gain control of a large amount of simultaneously originated clinical data. Other key limitations of the paper are also associated with the non-consideration of battery usage as well as data privacy. Future work could be extended to address these two limitations, too. Battery usage can be optimised by switching to a smarter learning operation that accomplishes convergence in less epoch, while blockchain can be used for data privacy.

## Data Availability

The study uses a hybrid dataset consisting of (i) primary data collected from voluntary participants using mobile devices and (ii) publicly available behavioral datasets used for model pre-training and benchmarking. The public dataset is available at: https://www.kaggle.com/competitions/emotion-prediction-based-on-passively-sensed-data/data. The Kaggle dataset was used only for pretraining and validation of feature extraction models, while the final IDMBD evaluation was conducted on the collected participant dataset.

## References

[1] Reddy, M. S., Rao, G. P., Kumar, S., Seshadri, V. & Prasant, P. 2022 Clinical characteristics, sociodemographic profile, and treatment pattern of bipolar disorder – A multicenter study from India. *Psychiatry Res. Commun.* **2**(2), 100039

[2] Narula, S., Pal, A., Reddy, M. S. & Mahajan, S. L. Research on clinical aspects of bipolar disorder: A review of Indian studies. *Indian J. Psychiatry*. **66** (5), 421–432 (2024). 38919568 10.4103/indianjpsychiatry.indianjpsychiatry_698_23 PMC11195747

[3] Hu, X. et al. Z 2022 Biomarkers and detection methods of bipolar disorder. *Biosens. Bioelectron.* **220**, 114842 36347076 10.1016/j.bios.2022.114842

[4] Straszek, S. P., Licht, R. W., Nielsen, R. E. & Vinberg, M. 2022 Bipolar depression, a challenge diagnostic and treatmentwise. *Ugeskr Laeger* **184**(14), V10210821–V10210821 . 35410656

[5] Baldessarini, R. J., Vázquez, G. H. & Tondo, L. Bipolar depression: a major unsolved challenge. *Int. J. Bipolar Disorders*. **8** (1), 1 (2020). 10.1186/s40345-019-0160-1 PMC6943098 31903509

[6] El-Mallakh, R. S. & Belmaker, R. H. *Bipolar-disorder. Tasman’s Psychiatry* (Springer International Publishing, 2023).

[7] Vogelbacher, C. et al. 2024 The German research consortium for the study of bipolar disorder (BipoLife): a quality assurance protocol for MR neuroimaging data. *Int. J. Bipolar Disorders* **12**(1), 33 10.1186/s40345-024-00354-7 PMC11427632 39327338

[8] Martini, J. et al. Early detection of bipolar disorders and treatment recommendations for help-seeking adolescents and young adults. *Int. J. Bipolar Disorders*. **9** (1), 23 (2021). 10.1186/s40345-021-00227-3 PMC8253866 34215910

[9] van Oosterzee, A. AI and mental health: evaluating supervised machine learning models trained on diagnostic classifications. *AI Soc.* **40** (6), 5077–5086 (2025). 40893146 10.1007/s00146-024-02012-z PMC12391223

[10] Khan, A. & Ali, R. Unraveling minds in the digital era: a review on mapping mental health disorders through machine learning techniques using online social media. *Social Netw. Anal. Min.* **14** (1), 78 (2024).

[11] Squires, M. et al. Deep learning and machine learning in psychiatry: a survey of current progress in depression detection, diagnosis and treatment. *Brain Inf.* **10** (1), 10 (2023). 10.1186/s40708-023-00188-6 PMC10123592 37093301

[12] Brossollet, I., Gallet, Q., Favre, P. & Houenou, J. 2023 Machine learning and brain imaging for psychiatric disorders: New perspectives. Neuromethods. Springer US. 37988551

[13] Romão, J., Melo, A., André, R. & Novais, F. Machine learning as a tool to find new pharmacological targets in mood disorders: A systematic review. *Curr. Treat. Options Psychiatry*. **11** (3), 241–264 (2024).

[14] Yashaswini, K. A. & Saxena, A. K. A novel predictive scheme for confirming state of bipolar disorder using recurrent decision tree. *Int. J. Adv. Comput. Sci. Appl.* **13**(2). (2022).

[15] Yashaswini, K. A. & Kasaragod, M. Framework towards critical event classification of bipolar disorder in Internet of Things ecosystem. *IAES Int. J. Artif. Intell.* **13**(3). (2024).

[16] Esposito, C. et al. Are there any differences in clinical and biochemical variables between bipolar patients with or without lifetime psychotic symptoms? *J. Clin. Med.* **12** (18), 5902 (2023). 37762843 10.3390/jcm12185902 PMC10531939

[17] Brancati, G. E. et al. Differential characteristics of bipolar I and II disorders: a retrospective, cross-sectional evaluation of clinical features, illness course, and response to treatment. *Int. J. Bipolar Disorders*. **11** (1), 25 (2023). 10.1186/s40345-023-00304-9 PMC10349025 37452256

[18] McIntyre, R. S. et al. 2022 The clinical characterization of the adult patient with bipolar disorder aimed at personalization of management. *World Psychiatry* **21**(3), 364–387 . 36073706 10.1002/wps.20997 PMC9453915

[19] Yashaswini, K. A. & Rao, S. *Bipolar-disorder: A pathway towards research progress in identification and classification. Advances in Intelligent Systems and Computing* (Springer International Publishing, 2020).

[20] Shen, G., Chen, H., Ye, X., Xue, X. & Tang, S. Machine learning-driven simplification of the hypomania checklist-32 for adolescent: a feature selection approach. *Int. J. Bipolar Disorders*. **12** (1), 42 (2024). 10.1186/s40345-024-00365-4 PMC11655905 39692968

[21] Sujatha, R., Tejesh, K., Krithi, H. & Shri, H. R. 2021 Detection of bipolar disorder using machine learning with MRI. International Symposium on Intelligent Control.

[22] Faurholt-Jepsen, M. et al. Voice analyses using smartphone-based data in patients with bipolar disorder, unaffected relatives and healthy control individuals. *Int. J. Bipolar Disorders*. **9** (1), 38 (2021). 10.1186/s40345-021-00243-3 PMC8632566 34850296

[23] Mohamed, E. S. et al. A hybrid mental health prediction model using Support Vector Machine, Multilayer Perceptron, and Random Forest algorithms. *Healthc. Analytics*. **3**, 100185 (2023).

[24] Saha, D. K. et al. Ensemble of hybrid model based technique for early detecting of depression based on SVM and neural networks. *Sci. Rep.* **14** (1), 25470 (2024). 39462047 10.1038/s41598-024-77193-0 PMC11513093

[25] Li, H. et al. Identification of bipolar disorder using a combination of multimodality magnetic resonance imaging and machine learning techniques. *BMC Psychiatry*. **20** (1), 488 (2020). 33023515 10.1186/s12888-020-02886-5 PMC7542439

[26] Nazari, M-J. et al. A machine learning approach for differentiating bipolar disorder type II and borderline personality disorder using electroencephalography and cognitive abnormalities. *PloS One* **19**(6), e0303699, (2024). 10.1371/journal.pone.0303699 PMC11192371 38905185

[27] Amanollahi, M. et al. Machine learning applied to the prediction of relapse, hospitalization, and suicide in bipolar disorder using neuroimaging and clinical data: A systematic review. *J. Affect. Disord*. **361**, 778–797 (2024). 38908556 10.1016/j.jad.2024.06.061

[28] Zakariah, M. & Alotaibi, Y. A. Unipolar and bipolar depression detection and classification based on actigraphic registration of motor activity using machine learning and uniform manifold approximation and projection methods. *Diagnostics* **13** (14), 2323 (2023). 37510067 10.3390/diagnostics13142323 PMC10377958

[29] Lin, E., Lin, C-H. & Lane, H-Y. Logistic ridge regression to predict bipolar disorder using mRNA expression levels in the N-methyl-D-aspartate receptor genes. *J. Affect. Disord*. **297**, 309–313 (2022). 34718036 10.1016/j.jad.2021.10.081

[30] Luján, M. A., Torres, A. M., Borja, A. L., Santos, J. L. & Sotos JM High-precise bipolar disorder detection by using radial basis functions based neural network. *Electronics* **11** (3), 343 (2022).

[31] Abdullah, M. & Negied, N. Detection and prediction of future mental disorder from social media data using machine learning, ensemble learning, and large language models. *IEEE Access.* **12**, 120553–120569 (2024).

[32] Belenguer-Llorens, A., Sevilla-Salcedo, C., Desco, M. & Soto-Montenegro, M. L. Gómez-Verdejo V A novel Bayesian Linear Regression model for the analysis of neuroimaging data. *Appl. Sci.* **12** (5), 2571 (2022).

[33] Busk, J. et al. Forecasting mood in bipolar disorder from smartphone self-assessments: Hierarchical Bayesian approach. *JMIR mHealth uHealth*. **8** (4), e15028 (2020). 32234702 10.2196/15028 PMC7367518

[34] Gilanie, G. et al. A robust method of bipolar mental illness detection from facial micro expressions using machine learning methods. *Intell. Autom. Soft Comput.* **39**(1), (2024 ).

[35] Zhu, Y. et al. Employing biochemical biomarkers for building decision tree models to predict bipolar disorder from major depressive disorder. *J. Affect. Disord*. **308**, 190–198 (2022). 35439462 10.1016/j.jad.2022.03.080

[36] Léger, M., Wolff, V., Kabuth, B., Albuisson, E. & Ligier, F. The mood disorder spectrum vs. schizophrenia decision tree: EDIPHAS research into the childhood and adolescence of 205 patients. *BMC Psychiatry*. **22** (1), 194 (2022). 35300648 10.1186/s12888-022-03835-0 PMC8932125

[37] Kamińska, D., Kamińska, O., Sochacka, M. & Sokół-Szawłowska, M. The role of selected speech signal characteristics in discriminating unipolar and bipolar disorders. *Sensors* **24** (14), 4721 (2024). 39066117 10.3390/s24144721 PMC11281009

[38] Llamocca, P., López, V., Santos, M., Čukić, M. & & Personalized characterization of emotional states in patients with bipolar disorder. *Mathematics* **9** (11), 1174 (2021).

[39] Liu, Y., Kang, K-D. & Doe, M. J. High-accuracy detection of depressed mood. *Technologies* **10** (6), 123 (2022).

[40] Metin, B. et al. The deep learning method differentiates patients with bipolar disorder from controls with high accuracy using EEG data. *Clin. EEG Neurosci.* **55** (2), 167–175 (2022). 36341750 10.1177/15500594221137234

[41] Huang, K-Y., Wu, C-H. & Su, M-H. Attention-based convolutional neural network and long short-term memory for short-term detection of mood disorders based on elicited speech responses. *Pattern Recogn.* **88**, 668–678 (2019).

[42] Cao, X., Zheng, S., Zhang, J., Chen, W. & Du, G. A hybrid CNN-Bi-LSTM model with feature fusion for accurate epilepsy seizure detection. *BMC Med. Inf. Decis. Mak.* **25**(1), 6, (2025). 10.1186/s12911-024-02845-0 PMC11706039 39762881

[43] Li, D., Liu, W. & Han H 2022 Mislabeled learning for psychiatric disorder detection. medRxiv.

[44] G’urpide, A. & Middleton M 2025 Mind the gaps: improved methods for the detection of periodicities in unevenly-sampled data. arXiv.

[45] Kim, R. H., Paulino, Y. C. & Kawabata, Y. Validating constructs of the depression, anxiety, and Stress Scale-21 and exploring health indicators to predict the psychological outcomes of students enrolled in the Pacific Islands Cohort of College Students. *Int. J. Environ. Res. Public. Health*. **21** (4), 509 (2024). 38673419 10.3390/ijerph21040509 PMC11050516

[46] Reynaud, E. et al. Validity and usage of the Seasonal Pattern Assessment Questionnaire (SPAQ) in a French population of patients with depression, bipolar disorders and controls. *J. Clin. Med.* **10** (9), 1897 (2021). 33925578 10.3390/jcm10091897 PMC8123881

[47] Ogunleye, B., Sharma, H. & Shobayo, O. Sentiment informed sentence BERT-ensemble algorithm for depression detection. *Big Data Cogn. Comput.* **8** (9), 112 (2024).

[48] Vázquez-Romero, A. Gallardo-Antolín A Automatic detection of depression in speech using ensemble Convolutional Neural Networks. *Entropy* **22** (6), 688 (2020). 33286460 10.3390/e22060688 PMC7517226

[49] Yan, K. et al. TCEDN: A lightweight time-context enhanced depression detection network. *Life* **14** (10), 1313 (2024). 39459613 10.3390/life14101313 PMC11509182

[50] Zou, L. et al. Mindfulness-based Baduanjin exercise for depression and anxiety in people with physical or mental illnesses: A systematic review and meta-analysis. *Int. J. Environ. Res. Public. Health*. **15(2)**, 321 (2018). 29439556 10.3390/ijerph15020321 PMC5858390

[51] Montes, J. M. et al. Assessment tool of bipolar disorder for primary health care: The SAEBD. *Int. J. Environ. Res. Public. Health*. **18** (16), 8318 (2021). 34444069 10.3390/ijerph18168318 PMC8392302

[52] Lin, Z., Wang, Y., Zhou, Y., Du, F. & Yang Y MLM-EOE: Automatic depression detection via sentimental annotation and multi-expert ensemble. *IEEE Trans. Affect. Comput.* 1–18 (2025) .

[53] Wang, Y., Lin, Z., Yang, C. & Zhou, Y. Yang Y Automatic depression recognition with an ensemble of multimodal spatio-temporal routing features. *IEEE Trans. Affect. Comput.* **16** (3), 1855–1872 (2025).

